# Observing others give & take: A computational account of bystanders’ feelings and actions

**DOI:** 10.1371/journal.pcbi.1010010

**Published:** 2022-05-02

**Authors:** Bastien Blain, Joseph Marks, Philipp Czech, Tali Sharot

**Affiliations:** 1 Affective Brain Lab, Experimental Psychology, University College London, London, United Kingdom; 2 Berlin School of Mind and Brain, Humboldt-Universität zu Berlin, Berlin, Germany; 3 The Max Planck UCL Centre for Computational Psychiatry and Ageing Research, University College London, London, United Kingdom; 4 Department of Brain and Cognitive Sciences, Massachusetts Institute of Technology, Boston, United State of America; Brain and Spine Institute (ICM), FRANCE

## Abstract

Social interactions influence people’s feelings and behavior. Here, we propose that a person’s well-being is influenced not only by interactions they experience themselves, but also by those they observe. In particular, we test and quantify the influence of observed selfishness and observed inequality on a bystanders’ feelings and non-costly punishment decisions. We developed computational models that relate others’ (un)selfish acts to observers’ emotional reactions and punishment decisions. These characterize the rules by which others’ interactions are transformed into bystanders’ reactions, and successfully predict those reactions in out-of-sample participants. The models highlight the impact of two social values—‘selfishness aversion’ and ‘inequality aversion’. As for the latter we find that even small violations from perfect equality have a disproportionately large impact on feelings and punishment. In this age of internet and social media we constantly observe others’ online interactions, in addition to in-person interactions. Quantifying the consequences of such observations is important for predicting their impact on society.

## Author summary

Social interactions can have a significant emotional impact on individuals. For example, being the recipient of generosity will likely make us feel good, while being the recipient of selfish behavior will likely make us feel bad. Here we tested the hypothesis that individuals’ feelings will be impacted by observing others interact, and that individuals may even choose to punish people who made selfish or inequal splits of resources. We recorded observers’ explicit affective reactions and non-costly punishment choices in response to other people’s decisions to allocate resources to themselves and another individual. Using a mathematical equation, we found that participants felt most negatively towards selfish allocators and punished them the most, which is a possible indicator of both selfishness aversion and inequality aversion. Intriguingly, participants felt more negatively towards generous allocators that gave more than half their share, and punished them more often, than those who split equally. Such behavior is consistent with inequality aversion. In fact, we find that even small deviations from equal splits resulted in especially large increases in observers’ negative feelings and punishments. This suggests that ‘pure equal split’ holds special status in the eye of the observer.

## Introduction

Humans are social animals. We live among, and interact with, other humans daily. Social interactions can have a significant emotional impact on individuals [1–5]. For example, being the recipient of generosity will likely make us feel good, while being the recipient of selfish behavior will likely make us feel bad. Because people care not only about their own well-being but also that of others [6–8], it is likely that individuals will be impacted not only by direct interactions, but also by observing others interact. In other words, people may feel good when observing others behave generously and feel bad when observing others behave selfishly, even if they are not the recipient of the behavior.

Here, we set out to characterize the computational rules by which observations are translated into feelings and action. This knowledge is important for two reasons. First, policymakers seeking to estimate the welfare effects of public-policy may wish to consider not only how a policy will impact people directly, but also how it may impact observing-third-parties indirectly [9,10]. For example, stringent anti-discrimination policies may benefit not only those vulnerable to discrimination but also third parties observing others being discriminated against. Second, it is theorized that feelings are important in governing choices [11]. Thus, if we are able to measure people’s feelings when observing others interact we may be able to generate that person’s utility function and use it to predict action. For example, predicting the likelihood that an observer will intervene when observing discrimination.

We pose that two dominant social values may play a role: ‘selfishness aversion’ and ‘inequality aversion’. Because people are averse to selfish behavior they may have a negative reaction when observing others allocate more to themselves than to others [12–14]. Conversely, they may have a positive reaction when observing others allocate less to themselves than to others. However, inequality aversion [15–17] may cause a negative reaction when observing an unequal distribution of resources, regardless of whether this allocation resulted from generosity or selfishness. By fitting computational models to participants’ responses, we can tease apart and quantify the effects of selfishness aversion and inequality aversion on observers’ feelings and actions.

We hypothesized that an observers’ affective response to observing (un)selfishness and (in)equity would be associated with their decisions to punish. However, if selfishness aversion and inequality aversion impact feelings and punishment to different degrees we may observe divergence between the two.

To test these hypotheses, we recorded observers’ explicit affective reactions and punishment choices in response to other people’s decisions to allocate resources to themselves and another individual. In contrast to most other studies examining third-party punishment decisions [12–14], we designed a task where punishment is non-costly. We did this for two reasons. First, non-costly punishment is ubiquitous in our modern world. For example, people rate and/or comment on other people’s behaviour anonymously all the time, providing numerous opportunities for non-costly punishment. Second, we were interested in how bystanders *feel* after observing others behave well or badly, without those feeling being influenced by the material loss of costly punishment. Affective reactions of third-party observers, as opposed to punishment decisions, have been understudied, despite being important for well-being.

We used a computational modeling approach to relate observers’ affective reactions and punishment choices to others’ behavior, creating what we refer to as a ‘feeling function’ and a ‘punishment function’. These functions allow for quantification and prediction of the influence of others’ behavior on bystanders’ feelings and action.

## Results

**Fig 1:**
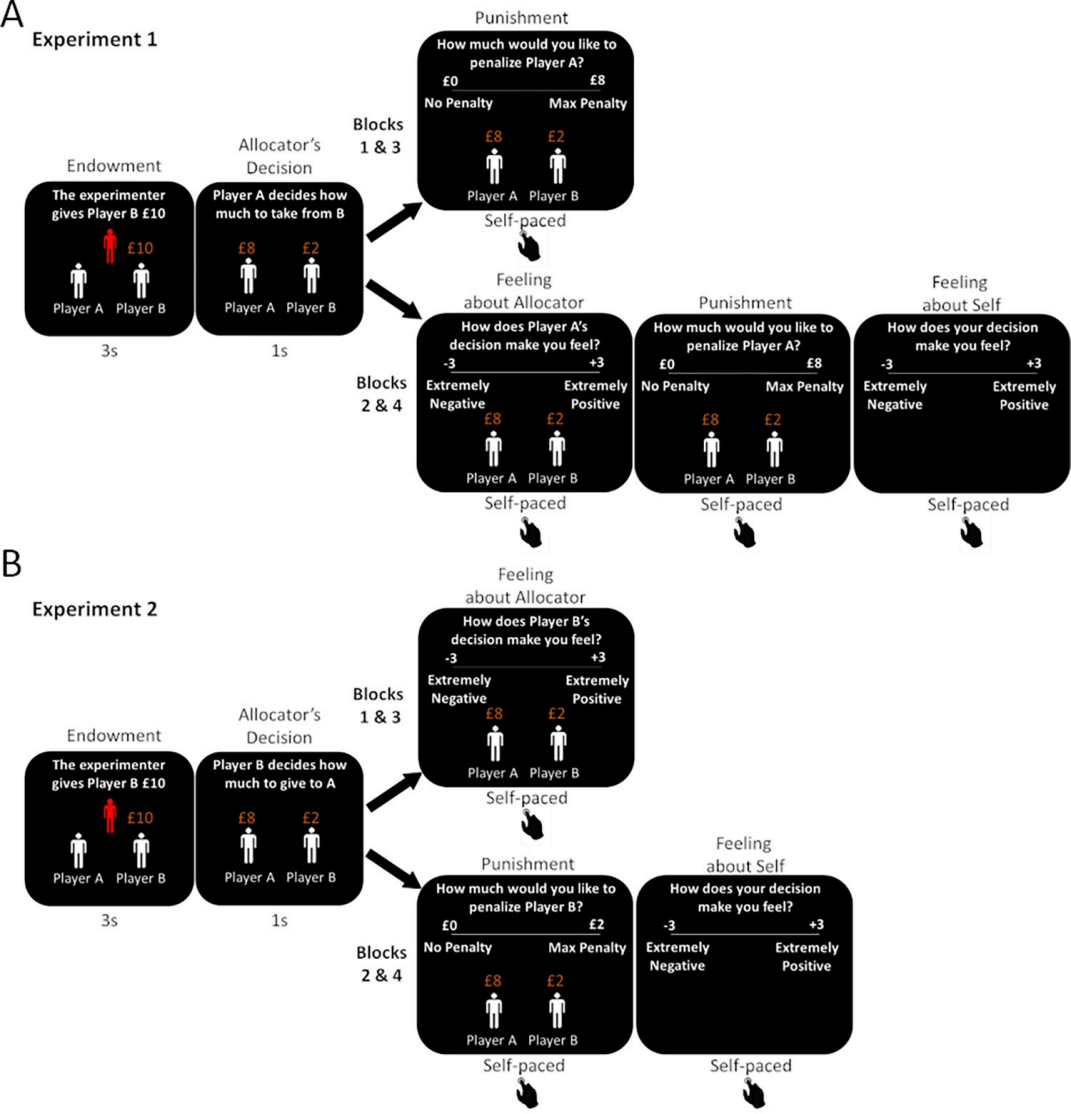
Task. Participants observed what they were led to believe were other participants’ resource allocation decisions. On each trial, the Experimenter gave a financial endowment (£1 to £15, step size = £1) to Player B. **Experiment 1 (A):** Player A, the allocator, could then take a portion of this money for themselves (10% to 100%, step size = 10%). In blocks 2 and 4 participants rated how they felt about the allocator’s decision. In all blocks, participants decided if and by how much to punish the allocator. In blocks 2 and 4 participants subsequently rated how they felt about their punishment decision. **Experiment 2 (B):** Player B was the allocator and could share a portion of their money with Player A (0% to 90%, step size = 10%). In blocks 1 and 3 participants rated how they felt about the allocator’s decision. In blocks 2 and 4 participants decided if and by how much to punish the allocator and then rated how they felt about their punishment decision.

We ran two experiments with a total of sixty-seven participants. On each of 240 trials a participant observed what they believed was another participant (the ‘allocator’, whom was said to differ on each trial) make decisions about how to divide a sum of money between themselves and what they believed to be a third participant (who differed on each trial). On some trials the participants had the opportunity to punish the allocator by giving some of the allocator’s money back to the experimenter and indicated how they felt about their punishment decision. On other trials the participant rated how they felt about the allocations. In Experiment 1 the allocating agents chose how much money to take from someone who had received an endowment from the experimenter (**Fig 1A**). In Experiment 2 the allocator was given an endowment and chose how much of it to give to another agent (**Fig 1B**). At the end of the study we asked participants if at any point throughout the experiment they thought the experimenter had deceived them in any way. 78% of subjects in Experimenter 1 and 77% participants in Experiment 2 said they did not suspect at any point that they were deceived by the experimenter in any way. We reanalysed all the data without those suspicious subjects and found the same results, which we report in **Tables E-G in S1 Text**.

### Observers’ affective responses are influenced by observed selfishness and inequality

**Fig 2:**
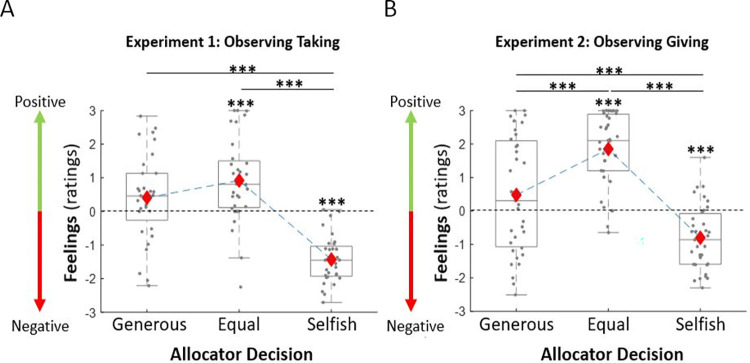
Feelings are negative when observing selfish behavior, positive when observing equal splits, and neutral when observing generosity. Observers report negative feelings when observing allocators act selfishly and positive feeling when observing equal splits. Surprisingly, feelings were not positive (nor negative) when observing generosity. This is true both in Experiment 1 (A, left panel) and Experiment 2 (B, right panel). The grey dots represent the mean feelings rating per allocator type for each participant. Red diamonds represent the average of these means. The box plots show the distribution of the participants’ mean feelings about each allocator: boxes indicate 25–75% interquartile range, whiskers extend from the first and third quartiles to most extreme data point within 1.5 × interquartile range, and the median is shown as a horizontal line within this box. *** p < 0.001.

First, we tested whether observers were negatively affected by observing others act selfishly—even though the selfish behavior was not directed towards them—and positively when observing others split the resources equally or act generously (**Fig 2**). A one-way repeated-measures ANOVA revealed a significant effect of the allocator type (selfish allocation, equal allocation, generous allocation) on feelings (Experiment 1: F(2,62) = 44.56, p < 0.001, η_p_^2^ = 0.59; Experiment 2: F(2,68) = 34.22, p < 0.001, η_p_^2^ = 0.50). Indeed, participants reported negative affect when watching the allocator take more than half the endowment (feeling rating significantly lower than zero: t(31) = -11.57, p < 0.001, d = -2.04) as well as keep more than half the endowment (t(34) = -4.94, p < 0.001, d = -0.84). When the allocator split the money equally the observers reported positive affect (feeling rating significantly greater than zero: Experiment 1: t(31) = 4.11, p < 0.001, d = 0.73; Experiment 2: t(34) = 9.84, p < 0.001, d = 1.66). Surprisingly, feelings were not significantly positive when observing allocators act generously—both when observing allocators give more than half the endowment (rating not significantly different from zero: t(34) = 1.63, p = 0.11, d = 0.28) and when observing them take less than half the endowment (t(31) = 1.82, p = 0.079, d = 0.32). In fact, when observing allocators act generously observers report feeling worse than when observing even splits (t(34) = -3.94, p < 0.001, d = -0.67) when generosity was due to giving more than half the endowment, and not different from equal splits (t(31) = -1.89, p = 0.068, d = -0.33) when generosity was due to taking less than half the endowment. As expected, participants reported feeling worse when observing selfishness than generosity (Experiment 1: t(31) = 6.84, p < 0.001, d = 1.21; Experiment 2: t(34) = 3.94, p < 0.001, d = 0.67) and equal splits (Experiment 1: t(31) = 9.53, *p* < 0.001, d = 1.68; Experiment 2: t(34) = 9.25, *p* < 0.001, d = 1.56).

What is underlying this surprising pattern of results? We hypothesized that if participants were averse to inequality, they may sometimes report feeling worse when observing unequal splits relative to even splits, even if unequal splits were a consequence of generous acts. At the same time aversion to selfishness will lead to more negative feelings to selfish acts than generosity even if inequality is the same in both conditions. To formally test this hypothesis, we quantified the influence of (un)selfishness and (in)equity on observers affect and characterized the computational rules by which features of observed acts are transformed into affective responses.

Modeling the data is essential to tease apart the effects of inequality aversion from that of selfishness aversion, as depending on the relative weights by which inequality and selfishness drive feelings their effect on ratings may cancel each other out. For example, in the case of generosity, inequality will trigger negative feelings but unselfishness positive feelings. By observing ratings alone, one may fail to detect these opposite drives (as is the case in Exp 1).

**Fig 3:**
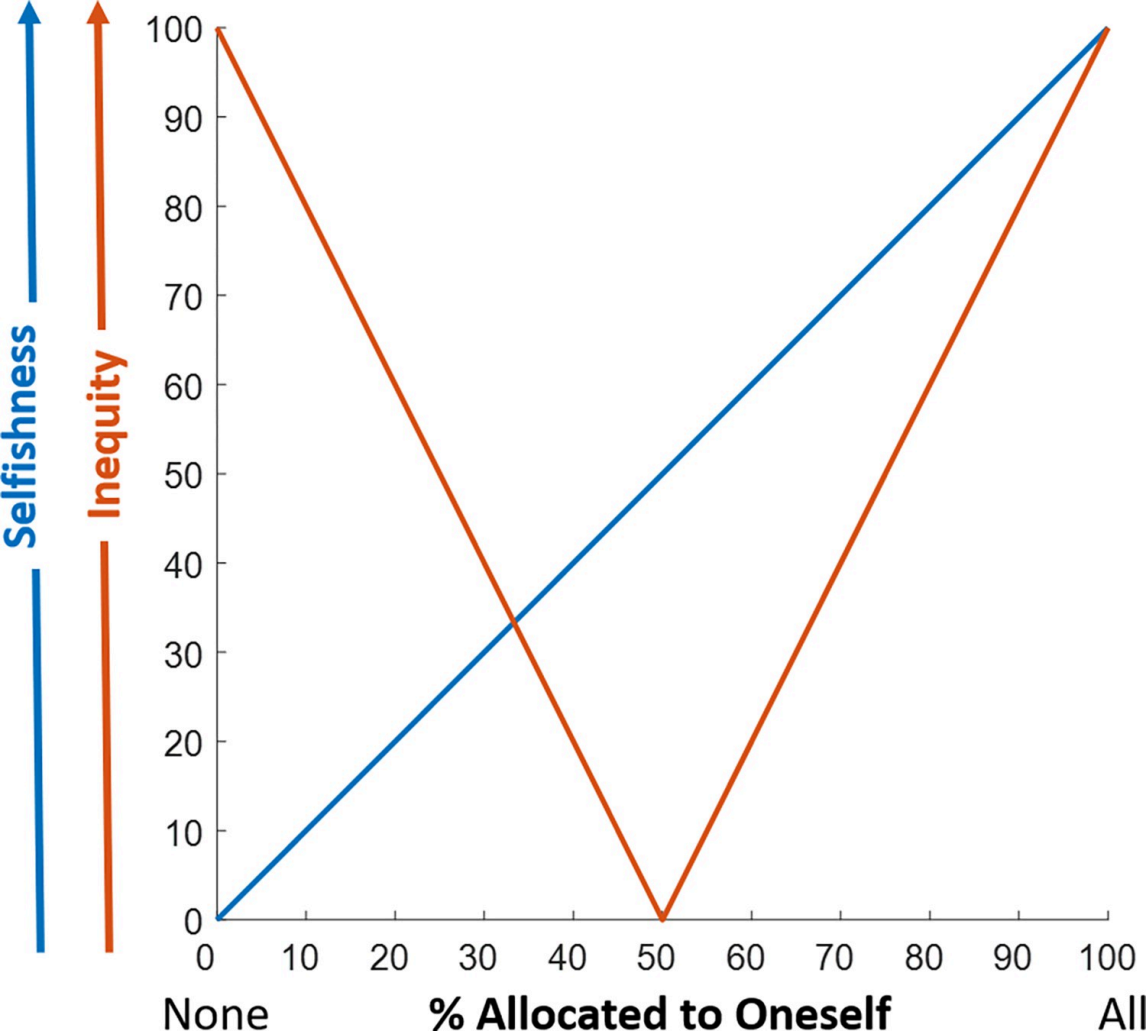
Operationalizing selfishness and inequality. Selfishness (blue line) is defined as the percentage of the endowment the allocator took/kept for themselves ranging from 0 (when the allocator is most generous, allocating nothing to themselves) to 100 (when the allocator is most selfish, allocating all to themselves). Inequality (orange line) is defined as the absolute difference between the percentages of the endowment each person is left with post allocation ranging from 0 (when the split was 50/50) to 100 (when one person receives all and the other none).

We operationalized selfishness (blue line, **Fig 3**) as the percentage of the endowment the allocator took/kept for themselves ranging from 0 (when the allocator is most generous, allocating nothing to themselves) to 100 (when the allocator is most selfish, allocating all to themselves). We operationalized inequality (orange line, **Fig 3**) as the absolute difference between the percentages of the endowment that each person is left with post allocation, ranging from 0 (when the split is 50/50) to 100 (when one person receives all and the other none).

**Fig 4:**
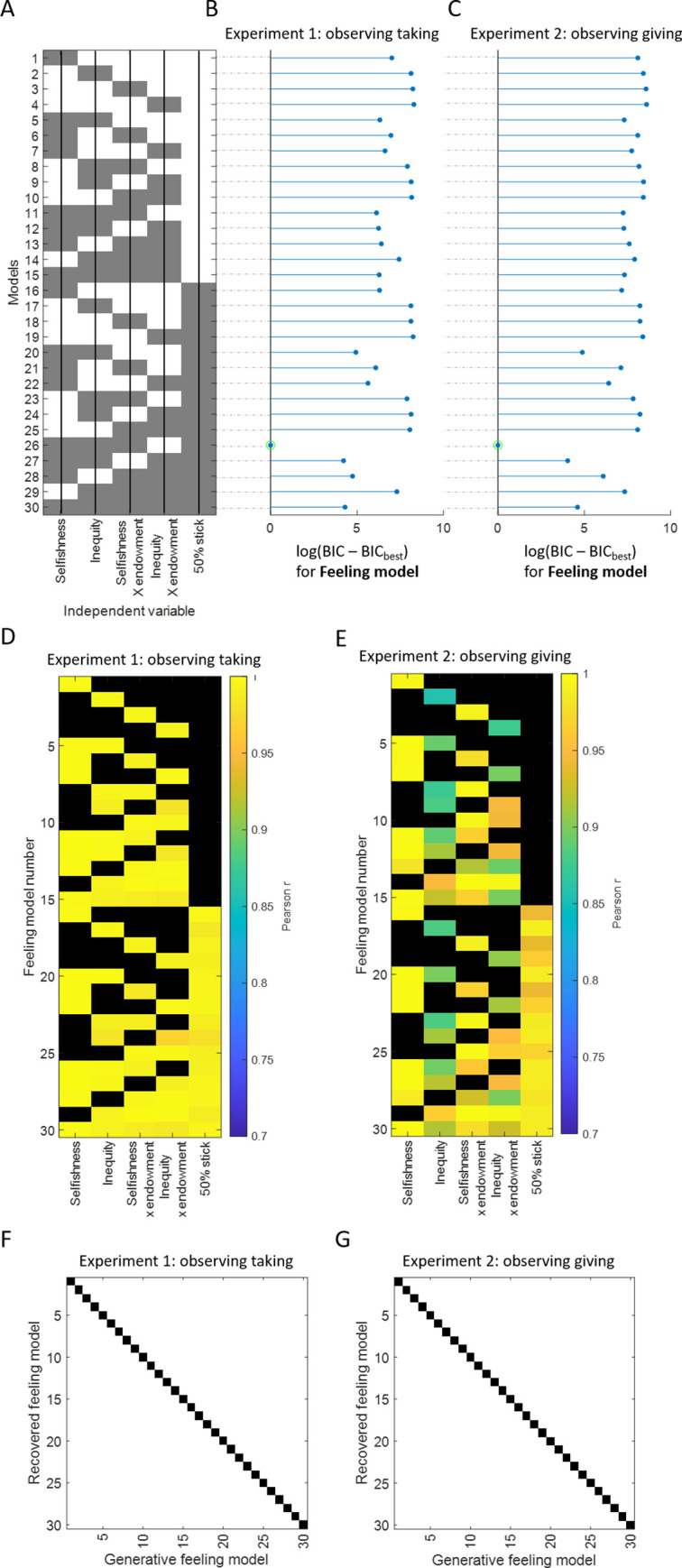
Feeling Models. (A) **Model specification.** Each row corresponds to a model, each column represents a parameter. Grey colour indicates that the parameter is included in the model. For example, the first row corresponds to model 1, which include selfishness only. Note that all the models also include a constant. (B, C) **Model selection.** Log delta BIC (difference between each model and the best model) is shown for (B) Experiment 1 & (C) Experiment 2. The green circle indicates the best model. Non-transformed ΔBIC between best model and second-best model > 50. (For additional information on model fit metric and ranking see **Table A in S1 Text**.) (D, E) **Parameter recovery.** Each row corresponds to a model and the columns represent the regressors. Coloured values correspond to the Pearson correlation r between the true parameters that generated the data and the estimated parameters in Experiment 1 (D) and 2 (E). The black colour is used when there is no parameter for this model. All the Pearson r are significant at p < 0.0001. (F, G) **Model recovery analysis**. The x-axis shows the model number which was used to simulate data and the y-axis the model number which was fit to the simulated data. The black color shows which model best fit the simulated data (compared to the second-best model using a ΔBIC > 30) for feelings simulated data in Experiment 1 (F) and Experiment 2 (G). The diagonal line indicates perfect model recovery. In other words, the model used to simulate the data was also the model that best fit that data. See methods for details.

**Fig 5:**
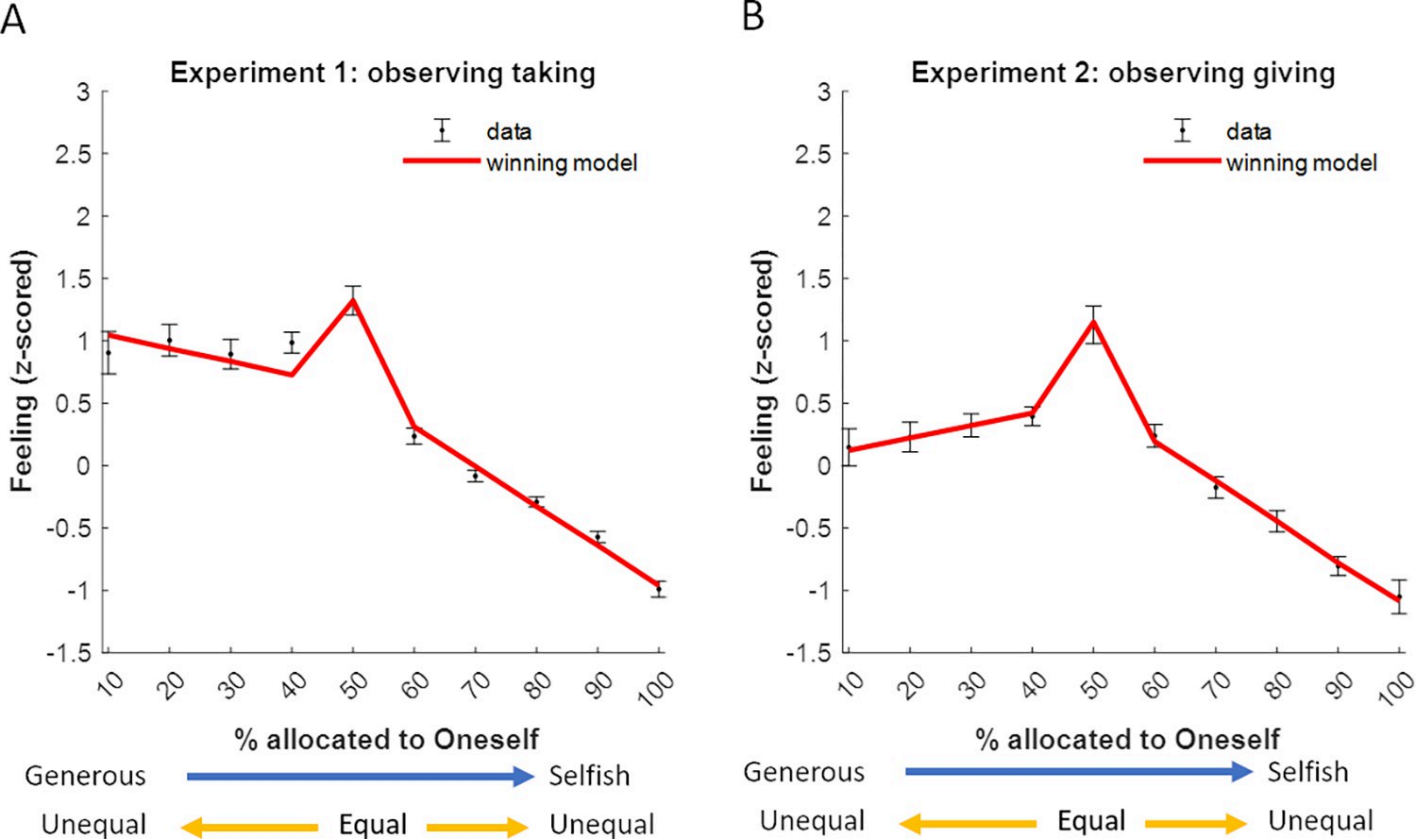
Modelling observers’ feelings as a function of observed selfishness and inequality. Plotted is the winning model (model 26) fit at the group-level for Experiment 1 (A,Feeling=1.7−1.86Selfishness−0.52Inequality−0.44Selfishness×Endowment+0.70evensplit) and Experiment 2 (B,Feeling=1.1−0.88Selfishness−1.1Inequality−0.42Selfishness×Endowment+0.63evensplit). Participants’ feelings ratings were z-scored before model-fitting to standardized responses. The predicted feelings from the model (red line) is overlaid on the mean observed feelings over all participants (black dots). Error bars represent SEM.

We then built computational models to quantify the impact of selfishness and inequality on observers’ feelings (**Fig 4)**. As selfishness or inequality may have particularly strong effects at high stake sizes, we also tested models that included an interaction between endowment and selfishness and/or between endowment and inequality. In addition, as can be observed in **Fig 5**, it seemed that subjects had a particularly strong positive reaction to a 50% split, suggesting that even small deviations from equality are perceived negatively. To capture this feature of the data we added a stick function at 50% split. The full model thus was as follows:

$$feelings=\beta_{0}+\beta_{1}selfishness+\beta_{2}inequality+\beta_{3}selfishness\times endowment+\beta_{4}inequality\times endowment+\beta_{5}\;50\%stick.$$ (1)

We estimated and compared all the nested models (all models included a constant term), resulting in 30 different models, summarised in **Fig 4A**. Each of these models were fit to each participant standardized feelings ratings separately. We validated our fit procedure and the use of BIC to accurately perform model selection through a parameter recovery analysis **Fig 4D and 4E** and a model recovery analysis, respectively (see **Fig 4F and 4G**).

The winning model (ΔBIC relative to the second-best model > 50; Exp 1: r^2^ = 0.67, Exp 2: r^2^ = 0.77; see **Fig 4B,** and **Table A in S1 Text**) was the following:

$$feelings=\beta_{0}+\beta_{1}selfishness+\beta_{2}inequality+\beta_{3}selfishness\times endowment+\beta_{5}\;50\%stick.$$ (2)

It included a selfishness parameter (Experiment 1: β = -1.86±0.28, CI = [-2.4, -1.3], t(31) = -6.7, p < 0.0001; Experiment 2: β = -0.88±0.32, CI = [-1.5, -0.76], t(34) = -2.8, p < 0.01), which indicates worse feeling as observed selfishness is greater; an interaction between selfishness and endowment (Experiment 1: β = -0.44±0.09, CI = [-0.61, -0.27], t(31) = -5.0, p < 0.00011; Experiment 2: β = -0.42±0.09, = [-0.61, -0.24], t(34) = -4.5, p < 0.001), which indicates the impact of selfishness on feelings is larger as endowment is larger (see also **Fig A in S1 Text**); an inequality parameter (Experiment 1: β = -0.52±0.17, CI = [-0.86, -0.18], t(31) = -3.2, p < 0.01; Experiment 2: β = -1.10±0.15, CI = [-1.35, -0.76], t(34) = -7.0, p < 0.0001), which indicates worse feeling as observed inequality is greater; and a parameter for an equal split ‘stick function’ (Experiment 1: β = 0.70±0.12, CI = [0.47, 0.92], t(31) = 5.9, p < 0.0001; Experiment 2: β = 0.63±0.16, CI = [0.32, 0.93], t(34) = 4.0, p < 0.001), which indicates feelings experience a large positive boost when splits are exactly equal (**Fig 5**). Removing suspicious participants from the analyses did not affect these results (see **Tables E-G in S1 Text** for details).

### Observer’s decisions to punish are a function of both selfishness aversion and inequality aversion

Thus far we report that the observers’ affective responses are increasingly negative as observed selfishness and inequality increases, and that large negative reactions are observed to even small deviations from pure equality. We next ask whether these same social values also drive observers’ punishment behavior.

**Fig 6:**
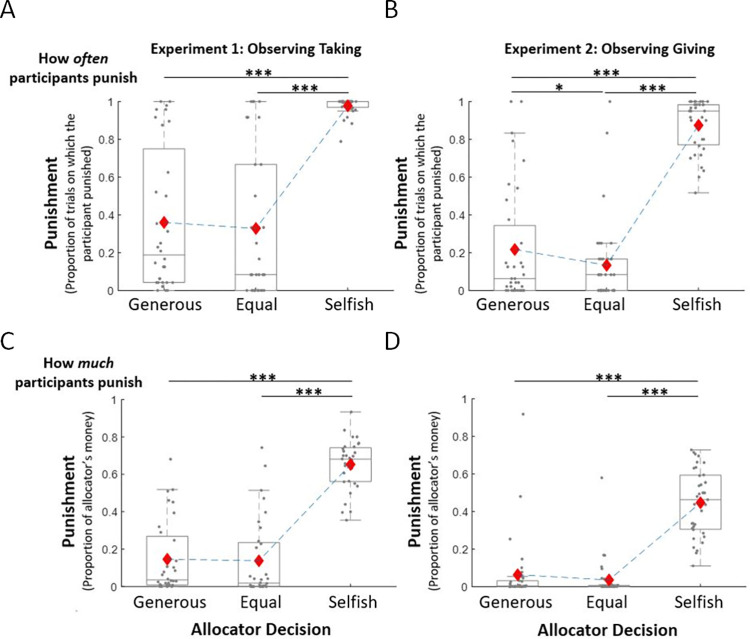
Observers’ Punishment. Participants punished selfish allocators more often (A & B) and more severely (C & D) than generous allocators and those who split resources equally in both Experiment 1 (A & C) and Experiment 2 (B & D). Surprisingly, participants also punished generous allocators more frequently than allocators who split the money equally in Experiment 2 (B & D) and punished them the same in Experiment 1 (A & C). Grey dots show the proportion of trials on which each participant punished each allocator (top panels) and the mean amount of punishment per allocator for each participant (bottom panels). Red diamonds represent the averages of these means. The box plots show the distribution of the participants’ mean punishment per allocator: boxes indicate 25–75% interquartile range, whiskers extend from the first and third quartiles to the most extreme data point within 1.5 × interquartile range, and the median is shown as a horizontal line within this box. * p < 0.05, *** p < 0.001.

The results of two one-way repeated-measures ANOVAs revealed that both the frequency of punishment (Experiment 1: F(2,62) = 83.30, p < 0.001, η_p_^2^ = 0.73. Experiment 2: F(2,68) = 169.11, p < 0.001, η_p_^2^ = 0.83) and the amount punished (Experiment 1: F(2,62) = 165.92, p < 0.001, η_p_^2^ = 0.84; Experiment 2: F(2,68) = 90.73, p < 0.001, η_p_^2^ = 0.73) vary according to the type of allocator being observed (see **Fig 6**). Observers punished selfish allocators more often than generous allocators (Experiment 1: selfish allocators: M = 0.98, SD = 0.05; generous allocators: M = 0.36, SD = 0.38; difference between the two: t(31) = 9.36, p < 0.001, d = 1.65. Experiment 2: selfish allocators: M = 0.87, SD = 0.14; generous allocators: M = 0.22, SD = 0.31; difference between the two: t(34) = 12.56, p < 0.001, d = 2.12) and more severely (Experiment 1: selfish allocators: M = 0.65, SD = 0.13; generous allocators: M = 0.15, SD = 0.20; difference between the two: t(31) = 13.86, p < 0.001, d = 2.45; Experiment 2: selfish allocators: M = 0.45, SD = 0.18; generous allocators: M = 0.06, SD = 0.18; difference between the two: t(34) = 8.82, p < 0.001, d = 1.49). They also punished selfish allocators more frequently than allocators who split the money equally (Experiment 1: equal allocators: M = 0.33, SD = 0.39; difference between the two: t(31) = 9.64, p < 0.001, d = 1.70. Experiment 2: equal allocators: M = 0.13, SD = 0.23; difference between the two: t(34) = 17.63, p < 0.001, d = 2.98) and more severely (Experiment 1: equal allocators: M = 0.14, SD = 0.21; difference between the two: t(31) = 13.81, p < 0.001, d = 2.44; Experiment 2: equal allocators: M = 0.04, SD = 0.10; difference between the two: t(34) = 11.54, p < 0.001, d = 1.94). Surprisingly, participants punished allocators who acted generously more often than allocators who split equally (t(34) = 2.28, p = 0.029, d = 0.39) when generosity was due to giving away more than half the endowment and no different than those who split equally (t(31) = 1.15, p = 0.26, d = 0.20) when generosity was due to taking away less than half the endowment. Moreover, punishment amount was the same for generous allocators and those who split equally (Experiment 1: t(31) = 0.41, p = 0.68, d = 0.07; Experiment 2: t(34) = 1.57, p = 0.13, d = 0.26). We note that there are also interesting individual differences, with some participants punish very often in response to generous splits, while others rarely do so.

**Fig 7:**
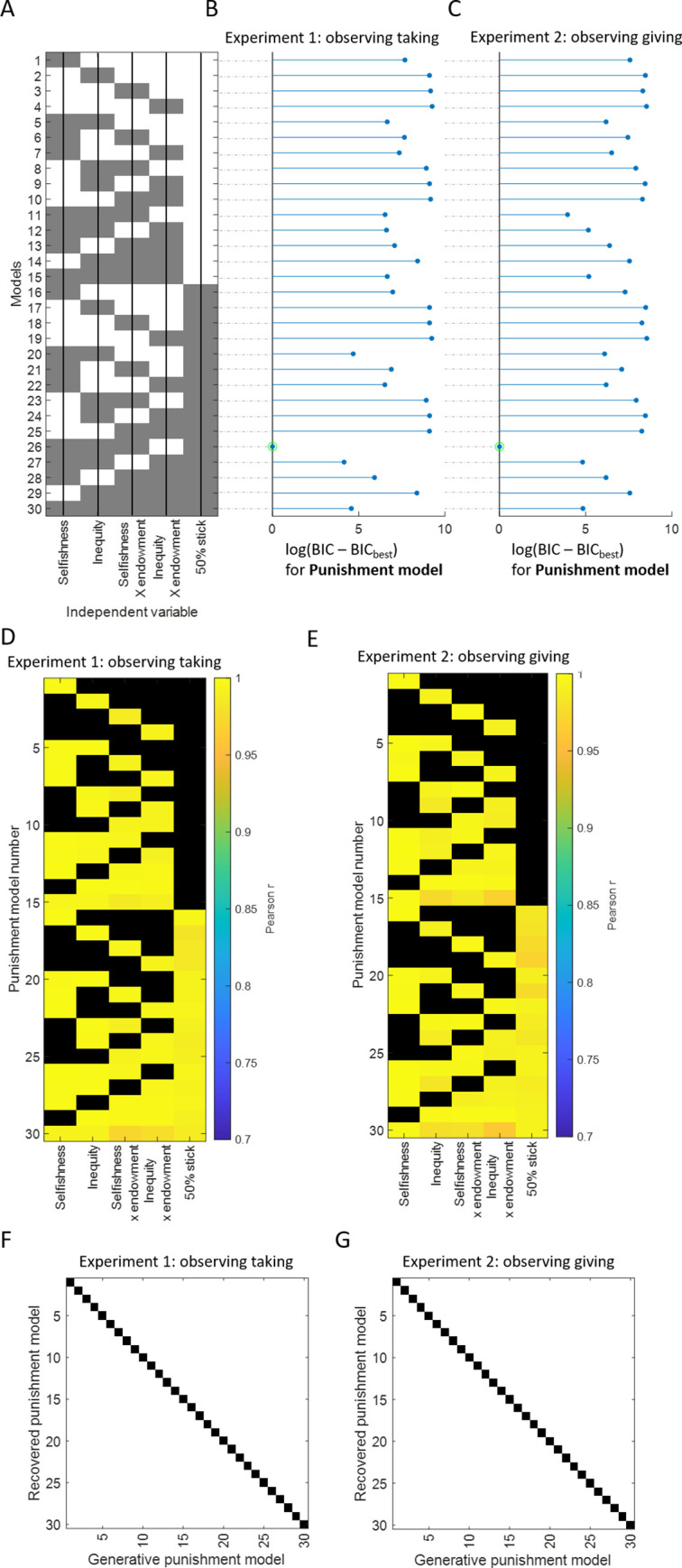
Punishment Model. **Model specification.** (A) **Model specification.** Each row corresponds to a model, each column represents a parameter. Grey colour indicates that the parameter is included in the model. For example, the first row corresponds to model 1, which include selfishness only. Note that all the models also include a constant. (B, C) **Model selection.** Log delta BIC (difference between each model and the best model) is shown for (B) Experiment 1 & (C) Experiment 2. The green circle indicates the best model. Non-transformed ΔBIC between best model and second-best model > 50. (For additional information on model fit metric and ranking see **Table B in S1 Text**.) **Parameter recovery.** Each row corresponds to a model and the columns represent the regressors. Coloured values correspond to the Pearson correlation r between the true parameters that generated the data and the estimated parameters in Experiment 1 (D) and 2 (E). The black colour is used when there is no parameter for this model. All the Pearson r are significant at p < 0.0001. **Model recovery analysis**. The x-axis shows the model number which was used to simulate data and the y-axis the model number which was fit to the simulated data. The black color shows which model best fit the simulated data (compared to the second-best model using a ΔBIC > 30) for feelings simulated data in Experiment 1 (F) and 2 (G) The diagonal line indicates perfect model recovery. In other words, the model used to simulate the data was also the model that best fit that data. See methods for details.

To formally examine whether selfishness aversion and/or inequality aversion underlie punishment decisions we fit each of the computational models described in **Fig 7A** to observers (standardized) punishment choices, just as we did for feelings. Fit is assessed using r^2^ and BIC. The greater the r^2^ and the lower the BIC, the better the fit. BIC, but not r^2^ penalizes for model complexity (see **Table A in S1 Text**). Modeling the data was essential to tease apart the effects of inequality aversion from that of selfishness aversion for the following reason: When an allocator is generous the two social values (inequality and selfishness) will impact a response in opposite directions—inequality aversion will lead to an adverse reaction but unselfishness to a positive reaction. Depending on the weights of these two factors, their effect on behavior may cancel each other out. Thus, if one was to observe behavior alone it would be difficult to identify these opposite drives. This is where computational models come in handy, allowing us to tease apart and quantify each effect.

**Fig 8:**
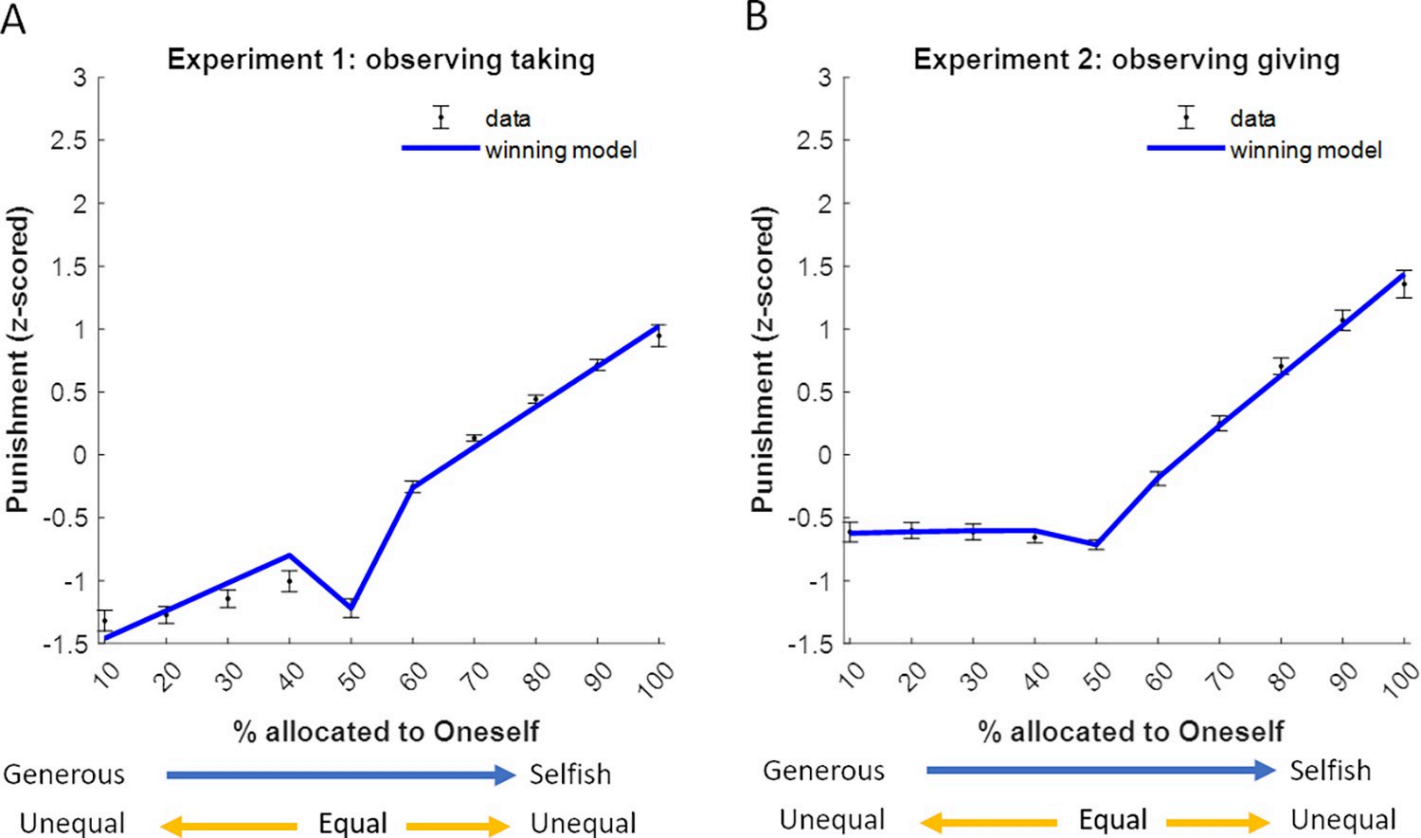
Observers’ punishment decisions reflect selfishness aversion and inequality aversion. Plotted are the winning models fit at the group-level for Experiment 1 (A,Punishment=−1.93+2.54Selfishness+0.25Inequality+0.30Selfishness×Endowment−0.64evensplit, left panel) and Experiment 2 (B,Punishment=−1.6+1.69Selfishness+0.99Inequality+0.68Selfishness×Endowment−0.12evensplit, right panel). Participants’ punishment choices were z-scored before model-fitting to standardize responses. The predicted punishment from the model (purple line) is overlaid on the mean observed punishment over all participants (purple dots). Error bars represent SEM.

Model comparison revealed that the same model that best explained feelings also best explained punishment decisions in both Experiment 1 and Experiment 2 (model 26; see **Fig 7B and 7C** and **Table B in S1 Text**). In both experiments the winning models indicate that greater punishment decisions were associated with greater observed selfishness (Experiment 1, β = 2.54±0.14, CI = [2.3, 2.8], t(31) = 18, p < 0.0001; Experiment 2, β = 1.69±0.20, CI = [1.3, 2.1], t(34) = 8.6, p < 0.0001), and greater inequality (Experiment 1: β = 0.25±0.12, CI = [0.021, 0.49], t(31) = 2.1, p = 0.041; Experiment 2, β = 0.99±0.10, CI = [0.8, 1.18], t(34) = 10, p < 0.0001). The stick function parameter for equal splits (Experiment 1: β = -0.64±0.08, CI = [-0.8, -0.49], t(31) = -8.3, p < 0.0001; Experiment 2 (β = -0.12±0.06, CI = [-0.26, 0.007], t(34) = -1.9, p = 0.07;) suggests that even small deviations from equal splits lead to relatively large increases in punishment (see **Fig 8**). Lastly, the interaction between observed selfishness and observed endowment (Experiment 1: β = 0.30±0.07, CI = [0.15, 0.44], t(31) = 4.1, p < 0.001, Experiment 2: β = 0.68±0.13, CI = [0.42, 0.93], t(34) = 5.2, p < 0.0001) suggests that selfishness effects punishments more as endowment increases (see **Fig A in S1 Text)**. Removing suspicious participants from the analyses did not affect these results (see **Table E-G in S1 Text** for details).

### Out-of-sample prediction

We next tested if the winning model’s predict new data that was not used in estimating it. In particular, we assessed whether the feelings function from one experiment was predictive of how observers felt in another experiment (and we did the same for punishment decisions). Because the experiments had slight differences between them (for example in one experiment the allocator took money and in the other gave it away) if the function generated by participant’s behavior in one experiment can predict their behavior in another, this will also suggest generalization across contexts.

We did this by entering the actual selfishness value, inequality value and endowment value on each trial from one experiment into the feelings function from the other. This generated a prediction of how each participant was feeling on each trial. To examine how good these predictions are we conducted a linear model for each subject predicting their data from these predictions. We also included an intercept. The average r^2^ across subjects was 0.50±0.03 for feelings. The slope was not different from 1 (M±SE = 0.94±0.06, t(66) = -0.94, p = 0.35) and intercept not different from 0 (M±SE = -0.012±0.035, t(66) = -0.35, p = 0.73). For punishments the average r^2^ across subjects was 0.64± 0.03(M±SE). The slope was not different from 1 (M±SE = 0.93±0.04, t(66) = -1.6, p = 0.12) and intercept not different from 0 (M±SE = 0.03±0.04, t(66) = 0.6, p = 0.54). Together, this suggests that the models are generalisable (see **Figs B and C in S1 Text**).

### Observers’ affective responses are related to their punishment

Given that both the feeling function and punishment functions were similar, we would expect a tight association between how a participant felt in response to the allocator’s decision and by how much they decided to punish him/her. In blocks 2 and 4 of Experiment 1 we recorded feelings and punishment on the same trials and can therefore test this relationship directly. Correlating each participant’s standardized punishment choices from these blocks with their standardized feelings ratings from these same blocks revealed that affective responses and punishment choices are tightly coupled (mean correlation coefficient across participants: r = -0.73, significantly different from zero: t(31) = -16.55, p < 0.001, d = -2.93). Indeed, participants punished more severely when they felt negatively, and less severely when they felt positively, about the allocator’s decision. Not only were feelings and punishment tightly correlated, but feelings predicted from the feeling model were also tightly correlated with observed punishments (Experiment 1: r = -0.78 ± 0.04, M ±SE; CI = [-0.85, -0.70], t(31) = -20.3, p < 0.0001; Experiment 2: r = -0.70 ± 0.04, M ±SE; CI = [-0.79, -0.61], t(34) = -16, p < 0.0001) and punishment predicted from the punishment model were tightly correlated with observed feeling (Experiment 1: r = -0.69 ± 0.05, M ±SE; CI = [-0.80, -0.59], t(31) = -14, p < 0.0001; Experiment 2: r = -0.57 ± 0.08, M ±SE; CI = [-0.72, -0.42], t(31) = -8.0, p < 0.0001).

### Differences in how selfishness aversion and inequality aversion impact feelings and actions

**Fig 9:**
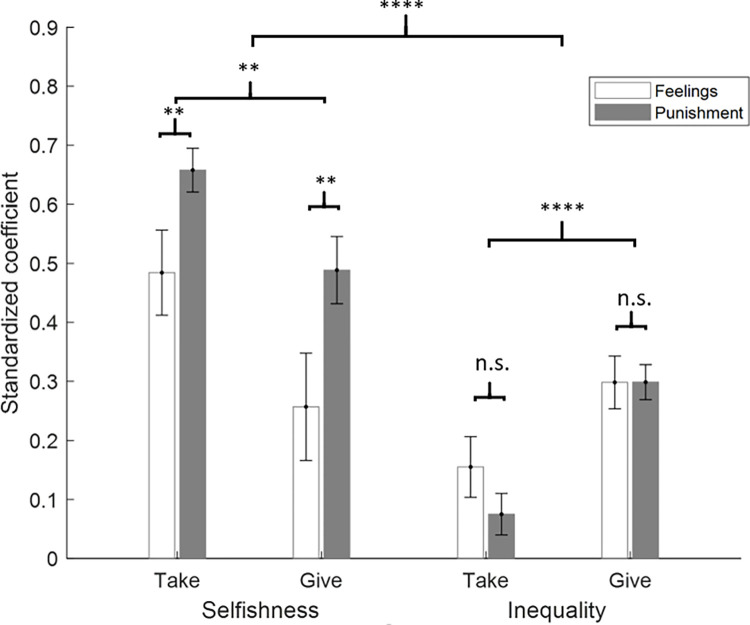
Transformed standardized coefficients (i.e. punishment coefficients are reversed for comparison) revealed a greater effect of selfishness on punishment than feelings, with no difference in the effect of inequality. In addition, reactions were more influenced by observing selfish taking than selfish giving, but vice versa when observing inequality. Furthermore, social values effects punishment more than feelings and selfishness effected responses more than inequality. Error bars correspond to the SEM.**p<0.01,****p<0.0001.

The above results suggest that feelings and punishments are strongly associated, yet not perfectly so. It is possible that part of the divergence is due to inequality and selfishness influencing action and affect to different extents and being weighted differently across contexts. To formally test this, we entered the standardized beta coefficient from the feelings and punishment models across all experiments into a repeated-measures ANOVA with (response: feelings/punishment) by social value (selfishness aversion/inequality aversion), as within-subject factors and experiment (experiment 1/experiment 2) as a between-subjects factor. As feelings and punishments are negatively associated (that is worse feelings lead to greater punishment) we first transformed the sign of the feeling estimates so that all the coefficients could be compared (see **Fig 9)**.

The analysis revealed an interaction between response type and social value (F(1,64) = 10.4, p < 0.01), which was characterized by a greater effect of selfishness on punishment than on feelings, (t(65) = -5.0, p < 0.001) with no difference in the effect of inequality (t = -1.5, p = 0.15). There was also an interaction between experiment and social value (F(1,64) = 11.9, p < 0.01), which was characterized by reactions being more influenced by observing selfish taking than selfish giving (t(130) = 2.65, p = 0.009), but vice versa when observing inequality (t(130) = -4.5 p < 0.0001). There was a main effects of response type (F(1,64) = 18.2, p < 0.0001) due to social values affecting punishment more than feelings. Finally, there was a main effect of social value (F(1,64) = 25.5, p < 0.0001), with selfishness affecting responses more than inequality. No other effects were significant.

## Discussion

In this study we characterize the rules by which other peoples’ interactions are transformed into bystanders’ reactions. Using computational models, we were able to tease apart the impact of two social values which are often confounded–selfishness aversion and inequality aversion. Moreover, we quantify their effect not only on punishment, but also on feelings, as we were interested in their impact on observers’ well-being.

We found that observers’ feelings and actions were governed by both ‘selfishness aversion’ (i.e., aversion to observing a person allocate more resources to themselves than to another) and ‘inequality aversion’ (i.e., aversion to observing a person divide resources unequally, even when inequality was the consequence of generosity). Participants felt most negatively towards selfish allocators and punished them the most, which is a possible indicator of both selfishness aversion and inequality aversion. Intriguingly, participants felt more negatively towards generous allocators that gave more than half their share, and punished them more often, than those who split equally. Such behavior is consistent with inequality aversion. In fact, we find that even small deviations from equal splits resulted in especially large increases in observers’ negative feelings and punishments. This suggests that ‘pure equal split’ hold special status in the eye of the observer.

A model that included both selfishness aversion and inequality aversion provided a better fit to participants’ feelings ratings and punishment choices than models that included only the former or latter. This was true both when participants observed the allocator take money and when they observed the allocator decide how much of the endowment to keep vs give. However, selfishness had a greater impact on reactions when observing taking than giving, while inequality had a greater impact on reactions when observing giving than taking. In fact, in the taking condition some participants severally punished even equal splits. Despite these differences, using a model generated from data gathered in one context (e.g., taking or giving) we were able to predict feelings and punishments in the other context.

Our model suggests that when the relative weight people place on inequality aversion is significantly greater than the weight they place on selfishness aversion, they will punish generous acts that create inequality. The findings help explain not only the behavior we report here, but also surprising behavioral phenomenon reported previously, such as antisocial punishment in public goods games (i.e. when people punish highly cooperative group members) [18–20] and do-gooder derogation (i.e. when people criticize those who engage in prosocial behavior) [21,22].

Unlike previous studies, (e.g. [12–17]), we designed the study such that punishment was not costly as we were interested in how feelings relate to actions when no hurdle was put in place to curb punishment. Non-costly punishment is common in real life (e.g., anonymously criticising people who broke social norms on social media or hurting their job prospects without them knowing) and yet is understudied. We suspect that our findings may have implications for real-world behavior, in the sense that both selfishness aversion and inequality aversion may help explain bystanders’ reactions in the ‘wild’. Such generalization requires empirical testing and future studies are also needed to examine the validity of our model in costly punishment situations. Nonetheless, there is evidence for the external validity of dictator games and third-party punishment games. For example, subjects’ behavior in laboratory dictator games is predictive of whether they will return a letter containing money that was ostensibly sent to the wrong address [23,24]. There are also correlations in behavior across different lab-based cooperation games—subjects’ who are prosocial in one cooperation game tend to be prosocial in other games–and correlations between behavior in such games and self-report measures of moral values [25], agreeableness and honesty/humility [26]. Similarly, punishment decisions in third-party punishment games are not only correlated with norm-enforcing punishment decisions in other games (e.g. second-party punishment and rejections of unfair offer in ultimatum games) [25] but are also correlated with individual differences in self-reported fairness and dominance preferences [27], dispositional sadism [28,29], and reduced empathic concern [30]. Punishment experiments also have explanatory power at the population level. Cultural variation in third-party punishment experiments is associated with factors that are relevant to the formation and maintenance of large-scale human cooperation. In particular, variation in altruistic punishment (i.e. costly punishment of selfishness) across different societies covaries with altruism in dictator games [31]. Populations that exhibit more altruistic punishment tend to show greater generosity, supporting macrosocial level models that predict altruistic punishment sustains norms of fairness and prosociality. Differences in anti-social punishment (i.e. costly punishment of generosity) across cultures, meanwhile, are associated with the World Values Survey’s measures of norms of civic cooperation and the extent to which people trust law enforcement institutions to be effective, fair, impartial and bound by the law in those countries [18]. Cultures with weak civic norms and a weak rule of law are more likely to engage in anti-social punishment, suggesting that vengeful or status-enhancing punishment is reduced in cultures where selfishness is viewed as unacceptable and institutionally discouraged.

In sum, we provide evidence that bystanders integrate multiple social values to govern decisions to change the status quo. The observed integration in bystanders is reminiscent of that reported previously in people who were choosing how much to directly give to others [15,17]. Our computation model, which describes this integration, makes it possible to successfully predict action and affect from observed (un)selfishness and (in)equity. Bystanders’ affective responses and subsequent actions may be adaptive in the long run as they could shape the behavior of other individuals in a group who may interact with the bystander in the future.

## Methods

### Ethics statement

The experiment was approved by the departmental ethics committee at University College London. Informed written consent was gained from participants.

All programming and data analyses were performed using MATLAB (The MathWorks, Natick, MA). Data and stimulus materials are publicly available on GitHub at https://github.com/BastienBlain/ObvservingOthersGiveAndTake.

### Experiment 1

#### Participants

Thirty-two participants completed the experiment (17 females and 15 males, aged 20–32 years M = 25.19, SD = 3.81). Sample size was equivalent to a previous study that used a within-subjects design to measure affective reactions when participants observed selfish and unselfish confederates receiving (painful) punishment [32]. One participant had to restart the task due to a technical error. Subjects were recruited from the UCL Division of Psychology and Language Sciences’ online subject pool. None were enrolled in economics and/or psychology courses. Participants were paid £12 for completing the experiment.

#### Procedure and task design

Participants came into the lab and were told that they would be observing simple distribution games between anonymous and varying pairs of players on Amazon’s Mechanical Turk. In reality, the other “players” were not participants but algorithms.

Instructions indicated that the computer will be connected with an online service that provides members the opportunity to earn money by participating in a simple online game.

We also stated that each trial corresponded to a different pair of participants (each interaction was therefore unique) and that everyone is anonymous (see **S1 Text** for the exact instructions).

The experiment consisted of 4 blocks of 60 trials each (**Fig 1**). On each trial participants observed as one player (Player B) was given a financial endowment (3sc), ranging from £1 to £15 (step size = £1). The other player (Player A, the allocator) then decided how much of Player B’s money to take for themselves (jittered duration 2-5sc). Participants were then shown the amount taken by Player A and the amount left for Player B (1sc). The amount taken varied on each trial from 10% to 100% of Player B’s money (step size = 10%).

On even blocks (i.e. blocks 2, 4) participants were asked to indicate “How does Player A’s decision make you feel?” (self-paced) on a visual analogue scale ranging from -3 (“extremely negative”) to 3 (“extremely positive”). In all blocks (i.e. 1, 2, 3, 4) they were then asked “By how much would you like to penalize Player A?” (self-paced) on a visual analogue scale ranging from £0 (“No penalty”) to the amount taken by Player A (e.g. £8; “Penalize by maximum amount”). Punishment was not costly to the participant. Neither did the participant nor Player B gain from the punishment. Rather the amount punished was deducted from Player’s A reward. On even blocks (i.e. blocks 2, 4) participants were then asked "How does your decision make you feel?” (self-paced) on a visual analogue scale ranging from -3 (“extremely negative”) to 3 (“extremely positive”). We chose to measure affective valence because it is considered a core component of affect, along with arousal [33–36]. Previous studies have demonstrated that self-report ratings of affective valence covary with physiological measures such as facial muscle activity (corrugator and zygomatic muscles) and heart rate acceleration [37], as well as neural activity [38].

The initial endowment was pseudo-randomized so that all 15 endowment amounts (£1-£15) were presented 4 times in each block for each participant. The amount taken by the allocator (10–100%, step-size = 10%) was also pseudo-randomized, independently of the endowment amount. On 9 trials the allocator took 100% of the other person’s endowment on that trial, on 9 trials they took 90%, on 9 trials they took 80%, on 9 trials they took 70%, on 9 trials they took 60%, on 3 trials they took 50%, on 3 trials they took 40%, on 3 trials they took 30%, on 3 trials they took 20%, on 3 trials they took 10%. This was done so that the players’ behavior would seem realistic.

Finally, participants completed a debriefing questionnaire (see **S1 Text**) consisting of a funneled debrief which gave them an opportunity to report any suspicions that the online players were bots. Excluding suspicious participants did not affect the main results reported below in either Experiment 1 or 2 (see **S1 Text and Table E-G in S1 Text**). They then provided demographic information and completed a series of standardized self-report questionnaires (see **S1 Text**). Upon completion participants were told the purpose of the study and informed that the other players were bots. The approximate duration of the experiment was 1.5 hours.

### Experiment 2

#### Participants

The recruitment procedure and compensation were the same as for Experiment 1. We aimed to recruit the same number of participants as in Experiment 1, accounting for a ten percent dropout rate. Thirty-five participants completed the experiment (22 females and 13 males, aged 18–61 years M = 27.91, SD = 11.18). One participant did not complete all of the trials in block one due to a technical error. The experiment was approved by the departmental ethics committee at University College London. Informed written consent was gained from participants.

#### Procedure and task design

The study instructions were the same as Experiment 1 except that in Experiment 1 the allocator decided how much money to *take* from the other player whereas in Experiment 2 the allocator decided how much money to *give* to the other player.

The experiment consisted of 4 blocks of 60 trials each (**Fig 1**). On each trial participants observed as one player (Player B) was given a financial endowment (3sc), ranging from £1 to £15 (step size = £1). Player B (the allocator) then decided how much of this money to give to Player A (jittered duration 2-5sc). Participants were then shown the amount given to Player A and the amount kept by Player B (1sc).

On odd blocks (i.e. blocks 1, 3) participants were asked to indicate “How does Player B’s decision make you feel?” on a visual analogue scale that ranged from -3 (“extremely negative”) to 3 (“extremely positive”; self-paced). On even blocks (i.e. 2, 4) participants were asked “By how much would you like to penalize Player B?” on a visual analogue scale that ranged from £0 (“No penalty”) to the amount kept by Player B (e.g. £2; “Penalize by maximum amount”; self-paced). As in Experiment 1, punishment was not costly to the participant. After indicating their punishment decisions in blocks 2 and 4, participants were then asked "How does your decision make you feel?” on a visual analogue scale that ranged from -3 (“extremely negative”) to 3 (“extremely positive”; self-paced).

The initial endowment the allocator received was pseudo-randomized, independently of the allocator’s decision, so that all 15 endowment amounts (£1-£15) were presented 4 times in each block. Contrary to Experiment 1, the distribution of allocator decisions in each block was uniform across all outcomes. Each possible allocator decision (i.e. allocator giving 0–90%, step-size = 10%) was observed 6 times in each block. That is, on 6 trials they gave 10%, on 6 trials they gave 20%, and so on so forth up to 90%.

Finally, participants completed the debriefing questionnaire (see **S1 Text**) consisting of a funneled debrief which gave them an opportunity to report any suspicions that the online players were bots. Removing suspicious participants from the analyses did not affect the main results (see **S1 Text and Tables E-G in S1 Text** for details). They also indicated, post-task, what they would have done if they had been in the role of Player B (the allocator) and what distribution decision they thought was fair. They then provided demographic information and completed a series of standardized self-report questionnaires (see **S1 Text**). Upon completion participants were told the purpose of the study and informed that the other players were bots. The approximate duration of the experiment was 1.5 hours.

### Data analyses experiment 1 & 2

#### Behavioral data analysis

For each participant, mean feelings ratings were calculated separately for trials in which the allocator allocated the resources selfishly, generously and equally. Selfish allocations were classified as those were the allocator took/kept more than half the endowment; generous allocations were classified as those were the allocator took/kept less than half the endowment; and equal allocations as those where the allocator split the endowment equally. For each type of allocator, we performed one-sample t-tests to assess whether feelings were significantly different from zero (i.e. significantly positive or negative). We then performed a one-way repeated-measures ANOVA for each experiment to assess whether participants felt more positively about some allocator decisions than others. When these ANOVAs revealed significant results, we followed up with pairwise comparisons.

Punishment was analyzed in terms of both the frequency and amount of punishment. To calculate the *frequency* of punishment, trials on which the participant chose not to punish (to allow for movement errors when using the slider, values representing less than 1% of the allocator’s money were categorized as non-punishment trials) were coded as 0 and trials on which the participant chose to punish any amount equal to or greater than 1% were coded 1. For each participant, we computed the mean proportion of trials on which they punished each type of allocator. We then performed a one-way repeated-measures ANOVA for each experiment to test whether participants punished some allocator types more frequently than others. When these revealed significant results, we followed up with pairwise comparisons.

To assess the impact of the allocator’s decision on the *amount* of punishment, we calculated the proportion of the allocator’s money that the participant deducted on each trial in blocks where punishment was recorded. We then computed the mean amount each participant punished each type of allocator. A one-way repeated-measures ANOVA was performed to test whether there were differences in how much participants punished the three types of allocators. When these revealed significant results, we followed up with pairwise comparisons.

To ensure that there were no significant differences in the mean level of endowment between the different allocator conditions, we performed one-way repeated-measures ANOVAs comparing the mean endowment amounts given to selfish, generous and equal allocators for each experiment. These analyses revealed that there were no significant differences in the endowment level between the three allocator conditions in either experiment (see **S1 Text** for statistics).

In half of the blocks in Experiment 1 (blocks 2 and 4), participants decided how much to punish allocators after indicating how they felt about the allocator’s decision. To ensure that participants punishment choices in these blocks were not influenced by carry over effects of the explicit feelings rating, we re-ran the frequency of punishment and amount of punishment analyses using only data from blocks where feelings were not recorded (i.e. blocks 1 and 3) and found that the results remained unchanged (see **S1 Text**).

### Computational modeling

To quantify the influence of (un)selfishness and (in)equity on observers’ feelings and punishment choices we fit models, using the glmfit function in Matlab (version 2021a), to the standardized feelings ratings and standardized punishment choices of each participant separately. We then report the average and the standard errors for each estimated parameter across all subjects and compare them to zero using a t-test, or to each other via independent or dependent t-test for post hoc pairwise comparison.

To ensure that the model and the parameters were recoverable, we performed model recovery and parameter recovery analyses following established procedures [39]. To test for parameter recovery, we first estimated the parameters for each participant from actual data. Then, we simulated data with each of the generative models using parameters estimated for each participant. To account for noise in the simulation, we computed the standard deviation of the residuals from the model at the individual level and then generated Gaussian noise with the same standard deviation using the MATLAB randn function and added that noise to generated ratings. We then fitted the simulated data and tested the correlation between the true parameters that generated the data to the fitted parameters.

The model space includes the nested versions of a model including selfishness, inequality, the interaction between endowment and selfishness and between endowment and inequality and a 50% stick function. All models included a constant term.

For each model, Bayesian information criterion (BIC) [40,41] and r^2^ were computed. Given that the models differed in their number of parameters, BIC (rather than r^2^), which penalizes models with additional parameters, was used to compare models. More specifically, we summed BIC across participants.

To ensure that the punishment models were not influenced by carry over effects in blocks where participants made punishment choices in the same trials they indicated how they felt about the allocator’s decision (blocks 2 and 4 in Exp 1), we re-fit each model to the standardized punishment choices made in Experiment 1 using only trials from blocks in which feelings were not recorded (i.e. blocks 1 and 3). Doing so did not affect the model comparison results (see **S1 Text**).

### Relating feelings to punishment

In Experiment 1 we had two blocks (blocks 2, 4) in which participants indicated how they felt in response to the allocator’s decision and then chose whether, and by how much, to punish the allocator. To investigate whether observers acted in accordance with their affective responses to selfishness and inequality when punishing we correlated each participant’s standardized (z-scored) punishment choices from these blocks with their standardized feelings ratings from the same blocks. We then assessed with a one-sample t-test whether across participants in each experiment the correlation coefficients were significantly different from zero. We also examined if the predicted feeling on a trial was related to how much participants punished on that trial by regressing each participant’s standardized punishment choices to the corresponding predicted feelings, and vice versa (that is, also tested whether each participants’ standardized feelings ratings was related to predicted punishment). We then report the corresponding correlation that we tested against 0 at the group level.

### Out-of-sample prediction

We assessed whether the feelings function from one experiment could predict how observers felt in the other experiment. Specifically, we used the feelings function from Experiment 1 to predict how participants felt on each trial in Experiment 2, and vice versa. This was done by entering the selfishness value, inequality value and endowment value of each trial into the respective feeling function with the group averaged estimates–which generated a prediction of how the participant was feeling on that trial. We next examined how closely the predicted feeling on a specific trial resembled the observed feeling on that trial by regressing a linear model to each participant’s standardized feelings ratings with the corresponding predicted feelings. We then report the r^2^ and assessed with a one-sample t-test whether across participants the slope was not different from 1, and the intercept not different from 0 at the group level.

We did the same as described above for punishment–that is, used the punishment function generated from one experiment to predict punishment on trials in the other experiment and then correlated each participant’s standardized punishment choices with the corresponding predicted punishment values. We then report the r^2^ and assess slope and intercept.

## Supporting information

S1 Text Supplementary information. Supplementary information include the model comparison metrics, task instructions, post-task questions, the comparison of the initial endowment between allocator conditions, the results after excluding punishment choices from blocks in Experiment 1 in which participants both indicated feelings ratings and made punishment choices, the results after excluding suspicious participants, and result suggesting that observers’ feel better about punishing allocators when those decisions align with their feelings about the allocation. **Table A. Feeling model comparison results.** Winning model indicated in BOLD. **Table B. Punishment model comparison results.** Winning model indicated in BOLD. **Table C**. **Mean endowment given to the allocator across conditions, block types and experiments. Table D**. **Punishment model comparison results when models are fit to data from blocks 1 and 3 only.** The winning model is indicated in BOLD. Note that it does not include an interaction term. **Table E**. **Feelings model results after exclusions.** The winning model (indicated in BOLD) is the same winning model (model 26) as in the main text. **Table F**. **Punishment model results after exclusions.** The winning model (indicated in BOLD) is the same winning model (model 26) as in the main text. **Table G**. **Results of the statistical tests reported in the main text after exclusion of suspicious participants. Fig A. Interaction between endowment and selfishness.** The x-axis represents the observed selfishness and the y-axis feelings (a and c) and punishment decisions (b and d). Each color represents an endowment level, from low (blue, 1) to high (dark red, 15). As can be observed selfishness effected feeling and punishments more for high endowments than low endowments. **Fig B. Cross-validation of the feeling model. Each scatter plot corresponds to a participant.** The y-axis corresponds to the actual data of one experiment and the x-axis to the predictions of that data from the winning model of the other experiment. The dashed black line corresponds to the y = x line, along which dots would ideally be aligned for perfect validation. The red is the regression line. **Fig C. Cross-validation of the punishment model. Each scatter plot corresponds to a participant.** The y-axis corresponds to the actual data of one experiment and the x-axis to the predictions of that data from the winning model of the other experiment. The dashed black line corresponds to the y = x line, along which dots would ideally be aligned for perfect validation. The red is the regression line.
(DOCX)
