# Supplementary material for: Observing others give & take: A computational account of bystanders’ feelings and actions
Source: PLoS Comput Biol. 2022 May 2;18(5):e1010010. doi: 10.1371/journal.pcbi.1010010 (PMC9098039; doi:10.1371/journal.pcbi.1010010)
Supplement: S1 Text — Supplementary information include the model comparison metrics, task instructions, post-task questions, the comparison of the initial endowment between allocator conditions, the results after excluding punishment choices from blocks in Experiment 1 in which participants both indicated feelings ratings and made punishment choices, the results after excluding suspicious participants, and result suggesting that observers’ feel better about punishing allocators when those decisions align with their feelings about the allocation. Table A. Feeling model comparison results. Winning model indicated in BOLD. Table B. Punishment model comparison results. Winning model indicated in BOLD. Table C. Mean endowment given to the allocator across conditions, block types and experiments. Table D. Punishment model comparison results when models are fit to data from blocks 1 and 3 only. The winning model is indicated in BOLD. Note that it does not include an interaction term. Table E. Feelings model results after exclusions. The winning model (indicated in BOLD) is the same winning model (model 26) as in the main text. Table F. Punishment model results after exclusions. The winning model (indicated in BOLD) is the same winning model (model 26) as in the main text. Table G. Results of the statistical tests reported in the main text after exclusion of suspicious participants. Fig A. Interaction between endowment and selfishness. The x-axis represents the observed selfishness and the y-axis feelings (a and c) and punishment decisions (b and d). Each color represents an endowment level, from low (blue, 1) to high (dark red, 15). As can be observed selfishness effected feeling and punishments more for high endowments than low endowments. Fig B. Cross-validation of the feeling model. Each scatter plot corresponds to a participant. The y-axis corresponds to the actual data of one experiment and the x-axis to the predictions of that data from the winning model of the other experiment. The dash [file pcbi.1010010.s001.docx]

**Supporting Information**

**Model comparison metrics**

|  | Feeling |  | Exp.1: Observing taking | | | Exp.2: Observing giving | | |
| --- | --- | --- | --- | --- | --- | --- | --- | --- |
| Model no. | Model specification | θ | r^2^ | BIC | rank | r^2^ | BIC | rank |
| 1 | y ~ 1 + Selfishness | 2 | 0.51 | 7362 | 16 | 0.43 | 8687 | 20 |
| 2 | y ~ 1 + Inequality | 2 | 0.29 | 9623 | 24 | 0.33 | 9914 | 26 |
| 3 | y ~ 1 + Selfishness x Endowment | 2 | 0.23 | 9994 | 28 | 0.22 | 10676 | 29 |
| 4 | y ~ 1 + Inequality x Endowment | 2 | 0.18 | 10230 | 30 | 0.2 | 10841 | 30 |
| 5 | y ~ 1 + Selfishness + Inequality | 3 | 0.61 | 6806 | 12 | 0.67 | 6912 | 11 |
| 6 | y ~ 1 + Selfishness + Selfishness x Endowment | 3 | 0.53 | 7306 | 15 | 0.45 | 8678 | 19 |
| 7 | y ~ 1 + Selfishness + Inequality x Endowment | 3 | 0.59 | 6999 | 14 | 0.59 | 7720 | 15 |
| 8 | y ~ 1 + Inequality + Selfishness x Endowment | 3 | 0.41 | 8986 | 20 | 0.5 | 8924 | 21 |
| 9 | y ~ 1 + Inequality + Inequality x Endowment | 3 | 0.31 | 9658 | 26 | 0.34 | 9962 | 28 |
| 10 | y ~ 1 + Selfishness x Endowment + Inequality x Endowment | 3 | 0.30 | 9750 | 27 | 0.39 | 9919 | 27 |
| 11 | y ~ 1 + Selfishness + Inequality + Selfishness x Endowment | 4 | 0.63 | 6709 | 8 | 0.69 | 6823 | 9 |
| 12 | y ~ 1 + Selfishness + Inequality + Inequality x Endowment | 4 | 0.63 | 6767 | 9 | 0.69 | 6879 | 10 |
| 13 | y ~ 1 + Selfishness + Selfishness x Endowment + Inequality x Endowment | 4 | 0.62 | 6856 | 13 | 0.64 | 7420 | 14 |
| 14 | y ~ 1 + Inequality + Selfishness x Endowment + Inequality x Endowment | 4 | 0.55 | 7939 | 18 | 0.6 | 8139 | 17 |
| 15 | y ~ 1 + Selfishness + Inequality + Selfishness x Endowment + Inequality x Endowment | 5 | 0.64 | 6784 | 10 | 0.69 | 6935 | 12 |
| 16 | y ~ 1 + Selfishness + 50% stick | 3 | 0.58 | 6796 | 11 | 0.65 | 6712 | 8 |
| 17 | y ~ 1 + Inequality + 50% stick | 3 | 0.31 | 9606 | 22 | 0.4 | 9100 | 22 |
| 18 | y ~ 1 + Selfishness x Endowment + 50% stick | 3 | 0.32 | 9618 | 23 | 0.45 | 9131 | 24 |
| 19 | y ~ 1 + Inequality x Endowment + 50% stick | 3 | 0.24 | 10063 | 29 | 0.34 | 9780 | 25 |
| 20 | y ~ 1 + Selfishness + Inequality + 50% stick | 4 | 0.65 | 6389 | 5 | 0.75 | 5558 | 4 |
| 21 | y ~ 1 + Selfishness + Selfishness x Endowment + 50% stick | 4 | 0.60 | 6687 | 7 | 0.67 | 6653 | 7 |
| 22 | y ~ 1 + Selfishness + Inequality x Endowment + 50% stick | 4 | 0.63 | 6529 | 6 | 0.72 | 6033 | 6 |
| 23 | y ~ 1 + Inequality + Selfishness x Endowment + 50% stick | 4 | 0.44 | 8914 | 19 | 0.58 | 7913 | 16 |
| 24 | y ~ 1 + Inequality + Inequality x Endowment + 50% stick | 4 | 0.33 | 9633 | 25 | 0.42 | 9120 | 23 |
| 25 | y ~ 1 + Selfishness x Endowment + Inequality x Endowment + 50% stick | 4 | 0.38 | 9395 | 21 | 0.54 | 8662 | 18 |
| 26 | **y ~ 1 + Selfishness + Inequality + Selfishness x Endowment + 50% stick** | **5** | **0.67** | **6251** | **1** | **0.77** | **5428** | **1** |
| 27 | y ~ 1 + Selfishness + Inequality + Inequality x Endowment + 50% stick | 5 | 0.66 | 6318 | 2 | 0.77 | 5483 | 2 |
| 28 | y ~ 1 + Selfishness + Selfishness x Endowment + Inequality x Endowment + 50% stick | 5 | 0.66 | 6365 | 4 | 0.74 | 5867 | 5 |
| 29 | y ~ 1 + Inequality + Selfishness x Endowment + Inequality x Endowment + 50% stick | 5 | 0.58 | 7736 | 17 | 0.68 | 6957 | 13 |
| 30 | y ~ 1 + Selfishness + Inequality + Selfishness x Endowment + Inequality x Endowment + 50% stick | 6 | 0.67 | 6324 | 3 | 0.77 | 5527 | 3 |

**Table A.** Feeling model comparison results. Winning model indicated in BOLD.

|  | Punishment |  | Exp.1: Observing taking | | | Exp.2: Observing giving | | |
| --- | --- | --- | --- | --- | --- | --- | --- | --- |
| Model no. | Model specification | θ | r^2^ | BIC | rank | r^2^ | BIC | rank |
| 1 | y ~ 1 + Selfishness | 2 | 0.63 | 12433 | 16 | 0.58 | 8025 | 18 |
| 2 | y ~ 1 + Inequality | 2 | 0.28 | 19242 | 22 | 0.27 | 10813 | 26 |
| 3 | y ~ 1 + Selfishness x Endowment | 2 | 0.24 | 19837 | 27 | 0.36 | 10163 | 24 |
| 4 | y ~ 1 + Inequality x Endowment | 2 | 0.15 | 20744 | 30 | 0.21 | 11135 | 29 |
| 5 | y ~ 1 + Selfishness + Inequality | 3 | 0.67 | 11050 | 9 | 0.69 | 6575 | 9 |
| 6 | y ~ 1 + Selfishness + Selfishness x Endowment | 3 | 0.64 | 12374 | 15 | 0.62 | 7803 | 15 |
| 7 | y ~ 1 + Selfishness + Inequality x Endowment | 3 | 0.65 | 11830 | 14 | 0.69 | 6761 | 12 |
| 8 | y ~ 1 + Inequality + Selfishness x Endowment | 3 | 0.42 | 17729 | 20 | 0.54 | 8806 | 19 |
| 9 | y ~ 1 + Inequality + Inequality x Endowment | 3 | 0.30 | 19270 | 23 | 0.31 | 10741 | 25 |
| 10 | y ~ 1 + Selfishness x Endowment + Inequality x Endowment | 3 | 0.26 | 19851 | 28 | 0.39 | 10084 | 23 |
| 11 | y ~ 1 + Selfishness + Inequality + Selfishness x Endowment | 4 | 0.68 | 10960 | 7 | 0.73 | 6144 | 2 |
| 12 | y ~ 1 + Selfishness + Inequality + Inequality x Endowment | 4 | 0.68 | 11018 | 8 | 0.72 | 6266 | 5 |
| 13 | y ~ 1 + Selfishness + Selfishness x Endowment + Inequality x Endowment | 4 | 0.67 | 11460 | 13 | 0.71 | 6684 | 11 |
| 14 | y ~ 1 + Inequality + Selfishness x Endowment + Inequality x Endowment | 4 | 0.57 | 14780 | 18 | 0.63 | 7972 | 16 |
| 15 | y ~ 1 + Selfishness + Inequality + Selfishness x Endowment + Inequality x Endowment | 5 | 0.68 | 11052 | 10 | 0.73 | 6270 | 6 |
| 16 | y ~ 1 + Selfishness + 50% stick | 3 | 0.66 | 11341 | 12 | 0.63 | 7549 | 14 |
| 17 | y ~ 1 + Inequality + 50% stick | 3 | 0.30 | 19277 | 24 | 0.29 | 10898 | 28 |
| 18 | y ~ 1 + Selfishness x Endowment + 50% stick | 3 | 0.31 | 19286 | 25 | 0.42 | 9950 | 22 |
| 19 | y ~ 1 + Inequality x Endowment + 50% stick | 3 | 0.19 | 20504 | 29 | 0.23 | 11213 | 30 |
| 20 | y ~ 1 + Selfishness + Inequality + 50% stick | 4 | 0.69 | 10383 | 4 | 0.70 | 6536 | 7 |
| 21 | y ~ 1 + Selfishness + Selfishness x Endowment + 50% stick | 4 | 0.68 | 11257 | 11 | 0.66 | 7291 | 13 |
| 22 | y ~ 1 + Selfishness + Inequality x Endowment + 50% stick | 4 | 0.68 | 10950 | 6 | 0.71 | 6578 | 10 |
| 23 | y ~ 1 + Inequality + Selfishness x Endowment + 50% stick | 4 | 0.43 | 17712 | 19 | 0.55 | 8871 | 20 |
| 24 | y ~ 1 + Inequality + Inequality x Endowment + 50% stick | 4 | 0.31 | 19301 | 26 | 0.32 | 10819 | 27 |
| 25 | y ~ 1 + Selfishness x Endowment + Inequality x Endowment + 50% stick | 4 | 0.33 | 19224 | 21 | 0.44 | 9924 | 21 |
| 26 | **y ~ 1 + Selfishness + Inequality + Selfishness x Endowment + 50% stick** | **5** | **0.70** | **10277** | **1** | **0.74** | **6093** | **1** |
| 27 | y ~ 1 + Selfishness + Inequality + Inequality x Endowment + 50% stick | 5 | 0.70 | 10339 | 2 | 0.73 | 6216 | 3 |
| 28 | y ~ 1 + Selfishness + Selfishness x Endowment + Inequality x Endowment + 50% stick | 5 | 0.70 | 10646 | 5 | 0.72 | 6575 | 8 |
| 29 | y ~ 1 + Inequality + Selfishness x Endowment + Inequality x Endowment + 50% stick | 5 | 0.60 | 14596 | 17 | 0.64 | 8005 | 17 |
| 30 | y ~ 1 + Selfishness + Inequality + Selfishness x Endowment + Inequality x Endowment + 50% stick | 6 | 0.71 | 10372 | 3 | 0.74 | 6219 | 4 |

**Table B.** Punishment model comparison results. Winning model indicated in BOLD.

**Task instructions: Experiment 1**

Welcome to this experiment!

At the end of the instructions, the computer will be connected with an online service that provides members the opportunity to earn money by participating in a simple online game.

There are three players on each trial: A, B and C. You are always player C.

At the beginning of a trial you will just be observing online players A and B.

Player B will receive between £1 and £15 from the experimenter. Then player A can take any amount of that money from player B.

There is nothing player B can do to influence how much player A takes them.

On each trial you will be playing with different online participants.

Everyone is anonymous.

**Task instructions: Experiment 2**

Welcome to this experiment!

At the end of the instructions, the computer will be connected with an online service that provides members the opportunity to earn money by participating in a simple online game.

There are three players on each trial: A, B and C. You are always player C.

At the beginning of a trial you will just be observing online players A and B.

Player A will receive between £1 and £15 from the experimenter. Then player A can share any amount of that money with player B.

There is nothing player B can do to influence how much player A shares with them.

On each trial you will be playing with different online participants.

Everyone is anonymous.

**Post-task questions: Experiment 1**

Participants were asked:

Were the instructions for all four sessions clear? (Yes/No). If No, please specify.

- 100% of the participants answered yes.

Were you at any stage confused or didn’t know what to do to perform the task? (Yes/No). If Yes, please specify.

- 100% of the participants answered no.

What do you think the purpose of this experiment is?

- 16 participants reported that they thought the purpose of the study was related to fairness judgements; 5 participants thought the purpose was to examine the link between emotion and decision making; 2 participants suggested that we were interested in both fairness and emotion; 3 participants said they didn’t know; and 6 participants provided some other response.

Did you participate in a similar version of this experiment (observation of distribution games with option to interact)? (Yes/No). If Yes, do you know what the purpose of the experiment was? If Yes, did your previous experience affect the decisions you made in today’s experiment? (Yes/No).

- 31 participants said ‘no’, 1 participant said ‘yes’. The participant who said ‘yes’ reported that they did not know the purpose of the experiment.

Have you read or learned about similar games previously? (Yes/No). If Yes, do you know which games? If Yes, did your knowledge affect the decisions you made in this game? (Yes/No).

- 30 participants said ‘no’, 2 participants said ‘yes’. The participants who said ‘yes’ could not name a specific game and neither thought their knowledge affected the decisions they made in the current experiment.

Overall, was the study what you had expected? If No, please explain why:

- 28 participants said ‘yes’, 4 participants said ‘no’. Explanations did not relate to any of the main research questions of this study.

Which of the following factors contributed to your decision to penalize player A (multiple

answers possible)?

| **Answer** | **Count** |
| --- | --- |
| Minimize Player A’s payoff | 8 |
| Have equal payoffs for Player A and Player B | 22 |
| Punish Player A | 14 |
| Have not penalized player A at all | 0 |
| Other | 4 |

Which of the following factors contributed to your motivation to penalize player A (multiple

answers possible)?

| **Answer** | **Count** |
| --- | --- |
| Empathy for player B | 17 |
| Aversion against player A | 9 |
| Care for fairness | 22 |
| Have not penalized player A at all | 0 |
| Other | 0 |

Overall, do you think your reasons to penalize player A changed throughout the task? (Yes/No). If Yes, please specify.

- 26 participants said ‘no’, 6 said ‘yes’. Those who said yes suggested that their reasons may have changed because they wanted to be fair on the given trial or because they were using a new strategy.

Did you, at any point throughout the experiment, think that the experimenter had deceived you in any way? (Yes/No). If Yes, please specify.

- 25 participants said ‘no’, 7 said ‘yes’. 6 out of the 7 who said ‘yes’ reported that they suspected that they were not playing the game online with real people.

Participants were asked if they had any final comments for the researchers. They then completed three standardized self-report questionnaires measuring social value orientation [1] (SVO-9; Mean = 12.00, SD = 4.03, Range = 9-19), fairness orientation [2] (6 items related to altruism and 2 catch questions from the MFQ-30-F; Mean = 23.00, SD = 3.44, Range = 15-30) and depression [3] (BDI-II; Mean = 3.81, SD = 5.31, Range = 0-26). Lastly, participants provided demographic information.

**Post-task questions: Experiment 2**

Participants were asked:

Were the instructions for all four sessions clear? (Yes/No). If No, please specify.

- 100% of the participants answered yes.

Were you at any stage confused or didn’t know what to do to perform the task? (Yes/No). If Yes, please specify.

- 33 of the participants answered ‘no’, 2 answered ‘yes’. One of those who answered ‘yes’ was unsure whether they were seeing the same players in every trial, while the other was unsure what was meant by the feelings questions.

What do you think the purpose of this experiment is?

- 16 participants reported that they thought the purpose of the study was related to fairness judgements and/or decision making; 6 participants thought the purpose was to examine the link between emotion and decision making; 2 participants suggested that we were interested in both fairness and emotion; 2 participants said they didn’t know; and 9 participants provided some other response.

Have you read or learned about similar experiments before? (Yes/No).

- 32 participants said ‘no’, 3 participants said ‘yes’.

Have you previously participated in a similar version of this experiment before? (Yes/No). If Yes, do you know which games? If Yes, Do you know what the purpose of the previous experiment you participated in was?

- 34 participants said ‘no’, 1 participant said ‘yes’. The participants who said ‘yes’ said they thought the previous study was related to autism.

Did you, at any point throughout the experiment, think that the experimenter had deceived you in any way? (Yes/No). If Yes, please specify.

- 27 participants said ‘no’, 8 said ‘yes’. 6 out of the 8 who said ‘yes’ reported that they suspected that they were not playing the game online with real people.

Imagine you were assigned to be Player A and given £10 by the experimenter. How much of that amount would you share with Player B?

- Mean = £4.03, SD = 1.76, Range = 0-5

What do you think is the fairest amount for Player A to give to Player B in this situation?

- Mean = £4.69, SD = .90, Range = 1-5

Did you penalize the Player As who kept all or most of the money for themselves? If Yes, what motivated your decision to penalize the Player As who kept all or most of the money for themselves?

- 27 participants said ‘yes’, 1 said ‘no’, 7 said ‘sometimes’. Of those who said ‘yes’, 15 cited fairness as the main motivator driving their punishment, 7 said selfishness or greediness, 4 said to create equality, 1 said both unfairness and selfishness, and 5 provided some other moral argument.

Did you penalize the Player As who gave away all or most of the money? If Yes, what motivated your decision to penalize the Player As who gave away all or most of the money?

- 6 participants said ‘yes’, 25 said ‘no’, 4 said ‘sometimes’. Of those who said ‘yes’, 6 said they punished because the allocator made a bad or stupid decision, 1 said that over generosity might make the co-player feel guilty, 1 said it was unfair, 1 cited equality, and 1 did not give a reason.

Participants then completed two standardized self-report questionnaires measuring fairness orientation [2] (6 items related to altruism and 2 catch questions from the MFQ-30-F; Mean = 31.69, SD = 4.97, Range = 14-38) and honesty-humility [4] (Mean = 36.09, SD = 6.36, Range = 18-46). Lastly, participants provided demographic information.

**Comparing the initial endowment between allocator conditions.**

We tested whether there were differences in the initial endowment between the three allocator types. One-way repeated-measures ANOVAs were performed, with the mean endowment for each type of allocation (selfish, equal, generous) entered as the dependent variable, separately for the feelings and punishment blocks from each experiment. These analyses revealed that there were no significant differences in the initial endowments between the conditions in the feelings blocks (Experiment 1: F(2,30) = 0.22, p = 0.81, η_p_^2^ = 0.01; Experiment 2: F(2,33) = 0.06, p = 0.94, η_p_^2^ = 0.004) or punishment blocks (Experiment 1: F(2,30) = 0.77, p = 0.47, η_p_^2^ = 0.05; Experiment 2: F(2,33) = 0.01, p = 0.99, η_p_^2^ < .001). This suggests that the results reported in the main text were not confounded by differences in the initial endowment between the allocation types. The mean and standard deviation of the initial endowments in the feelings and punishment blocks of experiments one and two are in **S1 Text Table C**.

| Experiment | Block Type | Allocation Type | Mean (SD) |
| --- | --- | --- | --- |
| 1 | Feelings | Selfish | 7.98 (.22) |
| 1 | Feelings | Equal | 7.88 (1.67) |
| 1 | Feelings | Generous | 8.10 (.88) |
| 1 | Punishment | Selfish | 7.97 (.25) |
| 1 | Punishment | Equal | 7.76 (2.02) |
| 1 | Punishment | Generous | 8.16 (.82) |
| 2 | Feelings | Selfish | 7.98 (.39) |
| 2 | Feelings | Equal | 8.05 (.94) |
| 2 | Feelings | Generous | 8.00 (.51) |
| 2 | Punishment | Selfish | 8.01 (.42) |
| 2 | Punishment | Equal | 8.00 (1.27) |
| 2 | Punishment | Generous | 7.99 (.51) |

**Table C**. Mean endowment given to the allocator across conditions, block types and experiments.

**Excluding punishment choices from blocks in Experiment 1 in which participants both indicated feelings ratings and made punishment choices.** In half of the blocks in Experiment 1 (blocks 2 and 4), participants decided how much to punish allocators after indicating how they felt about the allocator’s decision. To ensure that the results were not driven by carry over effects in these blocks, we re-ran the analyses where we used punishment data from Experiment 1 except only using data from blocks where feelings were not recorded (blocks 1 and 3).

As reported in the main text, one-way repeated-measures ANOVAs revealed that both the frequency of punishment (F(2,62) = 80.55, p < 0.001, η_p_^2^ = 0.72) and the amount punished (F(2,62) = 154.93, p < 0.001, η_p_^2^ = 0.83) vary according to the type of allocator being observed. Observers punished selfish allocators more often (t(31) = 9.32, p < 0.001, d = 1.67) and more severely (t(31) = 13.27, p < 0.001, d = 2.34) than generous allocators. They also punished selfish allocators more frequently (t(31) = 9.45, p < 0.001, d = 1.67) and more severely (t(31) = 13.44, p < 0.001, d = 2.38) than allocators who split the money equally. Punishment frequency was the same for generous allocators and those who split equally (t(31) = 1.37, p = 0.18, d = 0.24), as was punishment amount (t(31) = 0.67, p = 0.51, d = 0.12).

The best model, reported in **S1 Text Table D,** was a model including selfishness, inequality, and the 50% stick, but without an interaction term. This model indicates that greater punishment decisions were associated with greater observed selfishness (Experiment 1, β = 2.65±0.14, CI = [2.4, 2.9], t(31) = 19, P < 0.0001), and inequality (Experiment 1: β = 0.25±0.13, CI = [0.006, 0.50], t(31) = 2.0, P = 0.053). The stick function parameter for equal splits (Experiment 1: β = -0.64±0.08, CI = [-0.8, -0.48], t(31) = -7.9, P < 0.0001), suggest that even small deviations from equal splits lead to relatively large increases in punishment.

|  | Punishment |  | Exp.1: Observing taking | | |
| --- | --- | --- | --- | --- | --- |
| Model no. | Model specification | θ | r^2^ | BIC | rank |
| 1 | y ~ 1 + Selfishness | 2 | 0.62 | 6469 | 15 |
| 2 | y ~ 1 + Inequality | 2 | 0.28 | 9758 | 21 |
| 3 | y ~ 1 + Selfishness x Endowment | 2 | 0.22 | 10130 | 27 |
| 4 | y ~ 1 + Inequality x Endowment | 2 | 0.14 | 10561 | 30 |
| 5 | y ~ 1 + Selfishness + Inequality | 3 | 0.66 | 5833 | 6 |
| 6 | y ~ 1 + Selfishness + Selfishness x Endowment | 3 | 0.63 | 6512 | 16 |
| 7 | y ~ 1 + Selfishness + Inequality x Endowment | 3 | 0.64 | 6256 | 14 |
| 8 | y ~ 1 + Inequality + Selfishness x Endowment | 3 | 0.40 | 9139 | 19 |
| 9 | y ~ 1 + Inequality + Inequality x Endowment | 3 | 0.30 | 9839 | 23 |
| 10 | y ~ 1 + Selfishness x Endowment + Inequality x Endowment | 3 | 0.24 | 10200 | 28 |
| 11 | y ~ 1 + Selfishness + Inequality + Selfishness x Endowment | 4 | 0.67 | 5871 | 8 |
| 12 | y ~ 1 + Selfishness + Inequality + Inequality x Endowment | 4 | 0.67 | 5878 | 9 |
| 13 | y ~ 1 + Selfishness + Selfishness x Endowment + Inequality x Endowment | 4 | 0.66 | 6097 | 13 |
| 14 | y ~ 1 + Inequality + Selfishness x Endowment + Inequality x Endowment | 4 | 0.56 | 7750 | 18 |
| 15 | y ~ 1 + Selfishness + Inequality + Selfishness x Endowment + Inequality x Endowment | 5 | 0.68 | 5960 | 11 |
| 16 | y ~ 1 + Selfishness + 50% stick | 3 | 0.65 | 5958 | 10 |
| 17 | y ~ 1 + Inequality + 50% stick | 3 | 0.30 | 9837 | 22 |
| 18 | y ~ 1 + Selfishness x Endowment + 50% stick | 3 | 0.29 | 9918 | 25 |
| 19 | y ~ 1 + Inequality x Endowment + 50% stick | 3 | 0.18 | 10496 | 29 |
| 20 | **y ~ 1 + Selfishness + Inequality + 50% stick** | **4** | **0.68** | **5538** | **1** |
| 21 | y ~ 1 + Selfishness + Selfishness x Endowment + 50% stick | 4 | 0.66 | 5992 | 12 |
| 22 | y ~ 1 + Selfishness + Inequality x Endowment + 50% stick | 4 | 0.67 | 5845 | 7 |
| 23 | y ~ 1 + Inequality + Selfishness x Endowment + 50% stick | 4 | 0.42 | 9190 | 20 |
| 24 | y ~ 1 + Inequality + Inequality x Endowment + 50% stick | 4 | 0.31 | 9918 | 24 |
| 25 | y ~ 1 + Selfishness x Endowment + Inequality x Endowment + 50% stick | 4 | 0.32 | 9935 | 26 |
| 26 | y ~ 1 + Selfishness + Inequality + Selfishness x Endowment + 50% stick | **5** | 0.69 | 5568 | 2 |
| 27 | y ~ 1 + Selfishness + Inequality + Inequality x Endowment + 50% stick | 5 | 0.69 | 5576 | 3 |
| 28 | y ~ 1 + Selfishness + Selfishness x Endowment + Inequality x Endowment + 50% stick | 5 | 0.69 | 5719 | 5 |
| 29 | y ~ 1 + Inequality + Selfishness x Endowment + Inequality x Endowment + 50% stick | 5 | 0.59 | 7694 | 17 |
| 30 | y ~ 1 + Selfishness + Inequality + Selfishness x Endowment + Inequality x Endowment + 50% stick | 6 | 0.70 | 5662 | 4 |

**Table D**. Punishment model comparison results when models are fit to data from blocks 1 and 3 only. The winning model is indicated in BOLD. Note that it does not include an interaction term.

**Excluding suspicious participants.**

We re-ran all of the analyses reported in the main text after removing participants who reported in the debriefing that they thought they had been deceived (Exp 1 N removed = 7, Exp 2 N removed = 8). The results are reported in **S1 Text Tables E-G** and as can be seen the same wining model as in the main text (model 26) fits data best for both experiments, for both feeling and punishment.

|  | Feeling |  | Exp.1: Observing taking | | | Exp.2: Observing giving | | |
| --- | --- | --- | --- | --- | --- | --- | --- | --- |
| Model no. | Model specification | θ | r^2^ | BIC | rank | r^2^ | BIC | rank |
| 1 | y ~ 1 + Selfishness | 2 | 0.48 | 6064 | 16 | 0.41 | 7071 | 22 |
| 2 | y ~ 1 + Inequality | 2 | 0.31 | 7489 | 23 | 0.35 | 7735 | 26 |
| 3 | y ~ 1 + Selfishness x Endowment | 2 | 0.21 | 7950 | 29 | 0.21 | 8535 | 29 |
| 4 | y ~ 1 + Inequality x Endowment | 2 | 0.19 | 8033 | 30 | 0.20 | 8613 | 30 |
| 5 | y ~ 1 + Selfishness + Inequality | 3 | 0.60 | 5539 | 10 | 0.67 | 5574 | 12 |
| 6 | y ~ 1 + Selfishness + Selfishness x Endowment | 3 | 0.50 | 6035 | 15 | 0.43 | 7067 | 21 |
| 7 | y ~ 1 + Selfishness + Inequality x Endowment | 3 | 0.56 | 5741 | 14 | 0.57 | 6303 | 16 |
| 8 | y ~ 1 + Inequality + Selfishness x Endowment | 3 | 0.42 | 7065 | 20 | 0.51 | 6992 | 19 |
| 9 | y ~ 1 + Inequality + Inequality x Endowment | 3 | 0.32 | 7517 | 25 | 0.36 | 7790 | 27 |
| 10 | y ~ 1 + Selfishness x Endowment + Inequality x Endowment | 3 | 0.30 | 7695 | 27 | 0.37 | 7968 | 28 |
| 11 | y ~ 1 + Selfishness + Inequality + Selfishness x Endowment | 4 | 0.62 | 5467 | 7 | 0.68 | 5514 | 10 |
| 12 | y ~ 1 + Selfishness + Inequality + Inequality x Endowment | 4 | 0.62 | 5500 | 8 | 0.68 | 5548 | 11 |
| 13 | y ~ 1 + Selfishness + Selfishness x Endowment + Inequality x Endowment | 4 | 0.60 | 5609 | 11 | 0.63 | 6000 | 14 |
| 14 | y ~ 1 + Inequality + Selfishness x Endowment + Inequality x Endowment | 4 | 0.54 | 6356 | 18 | 0.60 | 6471 | 17 |
| 15 | y ~ 1 + Selfishness + Inequality + Selfishness x Endowment + Inequality x Endowment | 5 | 0.63 | 5534 | 9 | 0.68 | 5608 | 13 |
| 16 | y ~ 1 + Selfishness + 50% stick | 3 | 0.55 | 5703 | 13 | 0.64 | 5394 | 8 |
| 17 | y ~ 1 + Inequality + 50% stick | 3 | 0.33 | 7473 | 22 | 0.43 | 7042 | 20 |
| 18 | y ~ 1 + Selfishness x Endowment + 50% stick | 3 | 0.31 | 7672 | 26 | 0.45 | 7235 | 24 |
| 19 | y ~ 1 + Inequality x Endowment + 50% stick | 3 | 0.25 | 7903 | 28 | 0.35 | 7717 | 25 |
| 20 | y ~ 1 + Selfishness + Inequality + 50% stick | 4 | 0.63 | 5324 | 4 | 0.75 | 4382 | 4 |
| 21 | y ~ 1 + Selfishness + Selfishness x Endowment + 50% stick | 4 | 0.57 | 5651 | 12 | 0.66 | 5364 | 7 |
| 22 | y ~ 1 + Selfishness + Inequality x Endowment + 50% stick | 4 | 0.61 | 5459 | 6 | 0.71 | 4853 | 6 |
| 23 | y ~ 1 + Inequality + Selfishness x Endowment + 50% stick | 4 | 0.45 | 7020 | 19 | 0.59 | 6131 | 15 |
| 24 | y ~ 1 + Inequality + Inequality x Endowment + 50% stick | 4 | 0.35 | 7496 | 24 | 0.44 | 7082 | 23 |
| 25 | y ~ 1 + Selfishness x Endowment + Inequality x Endowment + 50% stick | 4 | 0.37 | 7466 | 21 | 0.53 | 6902 | 18 |
| 26 | **y ~ 1 + Selfishness + Inequality + Selfishness x Endowment + 50% stick** | **5** | **0.65** | **5240** | **1** | **0.76** | **4300** | **1** |
| 27 | y ~ 1 + Selfishness + Inequality + Inequality x Endowment + 50% stick | 5 | 0.65 | 5273 | 2 | 0.76 | 4328 | 2 |
| 28 | y ~ 1 + Selfishness + Selfishness x Endowment + Inequality x Endowment + 50% stick | 5 | 0.64 | 5365 | 5 | 0.74 | 4670 | 5 |
| 29 | y ~ 1 + Inequality + Selfishness x Endowment + Inequality x Endowment + 50% stick | 5 | 0.57 | 6238 | 17 | 0.68 | 5447 | 9 |
| 30 | y ~ 1 + Selfishness + Inequality + Selfishness x Endowment + Inequality x Endowment + 50% stick | 6 | 0.66 | 5305 | 3 | 0.77 | 4381 | 3 |

**Table E**. Feelings model results after exclusions. The winning model (indicated in BOLD) is the same winning model (model 26) as in the main text.

|  | Punishment |  | Exp.1: Observing taking | | | Exp.2: Observing giving | | |
| --- | --- | --- | --- | --- | --- | --- | --- | --- |
| Model no. | Model specification | θ | r^2^ | BIC | rank | r^2^ | BIC | rank |
| 1 | y ~ 1 + Selfishness | 2 | 0.60 | 10195 | 16 | 0.57 | 6448 | 18 |
| 2 | y ~ 1 + Inequality | 2 | 0.27 | 15129 | 22 | 0.28 | 8630 | 26 |
| 3 | y ~ 1 + Selfishness x Endowment | 2 | 0.23 | 15560 | 27 | 0.36 | 8124 | 24 |
| 4 | y ~ 1 + Inequality x Endowment | 2 | 0.14 | 16247 | 30 | 0.21 | 8912 | 29 |
| 5 | y ~ 1 + Selfishness + Inequality | 3 | 0.64 | 9180 | 9 | 0.69 | 5227 | 8 |
| 6 | y ~ 1 + Selfishness + Selfishness x Endowment | 3 | 0.62 | 10148 | 15 | 0.61 | 6261 | 15 |
| 7 | y ~ 1 + Selfishness + Inequality x Endowment | 3 | 0.63 | 9760 | 14 | 0.68 | 5438 | 12 |
| 8 | y ~ 1 + Inequality + Selfishness x Endowment | 3 | 0.40 | 13999 | 20 | 0.54 | 7050 | 19 |
| 9 | y ~ 1 + Inequality + Inequality x Endowment | 3 | 0.29 | 15136 | 23 | 0.31 | 8587 | 25 |
| 10 | y ~ 1 + Selfishness x Endowment + Inequality x Endowment | 3 | 0.25 | 15561 | 28 | 0.40 | 8054 | 23 |
| 11 | y ~ 1 + Selfishness + Inequality + Selfishness x Endowment | 4 | 0.66 | 9109 | 6 | 0.72 | 4889 | 2 |
| 12 | y ~ 1 + Selfishness + Inequality + Inequality x Endowment | 4 | 0.65 | 9140 | 8 | 0.72 | 4986 | 5 |
| 13 | y ~ 1 + Selfishness + Selfishness x Endowment + Inequality x Endowment | 4 | 0.64 | 9454 | 13 | 0.70 | 5357 | 11 |
| 14 | y ~ 1 + Inequality + Selfishness x Endowment + Inequality x Endowment | 4 | 0.55 | 11917 | 18 | 0.63 | 6378 | 16 |
| 15 | y ~ 1 + Selfishness + Inequality + Selfishness x Endowment + Inequality x Endowment | 5 | 0.66 | 9185 | 10 | 0.73 | 4988 | 6 |
| 16 | y ~ 1 + Selfishness + 50% stick | 3 | 0.64 | 9394 | 12 | 0.62 | 6078 | 14 |
| 17 | y ~ 1 + Inequality + 50% stick | 3 | 0.29 | 15154 | 25 | 0.29 | 8693 | 28 |
| 18 | y ~ 1 + Selfishness x Endowment + 50% stick | 3 | 0.30 | 15149 | 24 | 0.41 | 7967 | 22 |
| 19 | y ~ 1 + Inequality x Endowment + 50% stick | 3 | 0.19 | 16066 | 29 | 0.22 | 8980 | 30 |
| 20 | y ~ 1 + Selfishness + Inequality + 50% stick | 4 | 0.66 | 8668 | 3 | 0.70 | 5199 | 7 |
| 21 | y ~ 1 + Selfishness + Selfishness x Endowment + 50% stick | 4 | 0.65 | 9335 | 11 | 0.65 | 5865 | 13 |
| 22 | y ~ 1 + Selfishness + Inequality x Endowment + 50% stick | 4 | 0.66 | 9113 | 7 | 0.70 | 5296 | 10 |
| 23 | y ~ 1 + Inequality + Selfishness x Endowment + 50% stick | 4 | 0.42 | 13989 | 19 | 0.55 | 7100 | 20 |
| 24 | y ~ 1 + Inequality + Inequality x Endowment + 50% stick | 4 | 0.30 | 15160 | 26 | 0.32 | 8646 | 27 |
| 25 | y ~ 1 + Selfishness x Endowment + Inequality x Endowment + 50% stick | 4 | 0.32 | 15109 | 21 | 0.44 | 7947 | 21 |
| 26 | **y ~ 1 + Selfishness + Inequality + Selfishness x Endowment + 50% stick** | **5** | **0.68** | **8589** | **1** | **0.73** | **4853** | **1** |
| 27 | y ~ 1 + Selfishness + Inequality + Inequality x Endowment + 50% stick | 5 | 0.68 | 8622 | 2 | 0.73 | 4951 | 4 |
| 28 | y ~ 1 + Selfishness + Selfishness x Endowment + Inequality x Endowment + 50% stick | 5 | 0.67 | 8849 | 5 | 0.71 | 5270 | 9 |
| 29 | y ~ 1 + Inequality + Selfishness x Endowment + Inequality x Endowment + 50% stick | 5 | 0.57 | 11789 | 17 | 0.64 | 6405 | 17 |
| 30 | y ~ 1 + Selfishness + Inequality + Selfishness x Endowment + Inequality x Endowment + 50% stick | 6 | 0.68 | 8672 | 4 | 0.74 | 4950 | 3 |

**Table F**. Punishment model results after exclusions. The winning model (indicated in BOLD) is the same winning model (model 26) as in the main text.

| **Effect / Statistical Test** | **Result after removing suspicious participants** |
| --- | --- |
| **Observers’ affective responses are influenced by observed selfishness and inequality** | |
| Repeated-measures ANOVA: amount of punishment | Experiment 1: F(2,48) = 31.37, p < 0.001, η_p_^2^ = 0.57.  Experiment 2: F(2,52) = 26.67, p < 0.001, η_p_^2^ = 0.51. |
| One-sample t-test comparing feelings for selfish allocator to zero | Experiment 1: t(24) = −10.43, p < 0.001, d = -2.09.  Experiment 2: t(26) = -3.87, p < 0.001, d = -0.74. |
| One-sample t-test comparing feelings for equal allocator to zero | Experiment 1: t(24) = 3.37, p = 0.003, d = 0.67.  Experiment 2: t(26) = 9.29, p < 0.001, d = 1.79. |
| One-sample t-test comparing feelings for generous allocator to zero | Experiment 1: t(24) = 0.96, p = 0.35, d = 0.19.  Experiment 2: t(26) = 1.64, p = 0.11, d = .32. |
| Paired-samples t-test comparing generous and equal allocators | Experiment 1: t(24) = -1.97, p = 0.061, d = -0.39. Experiment 2: t(26) = -3.33, p = 0.003, d = 0.64. |
| Paired-samples t-test comparing generous and selfish allocators | Experiment 1: t(24) = 5.70, p < 0.001, d = 1.14; Experiment 2: t(26) = 3.49, p = 0.002, d = 0.67. |
| Paired-samples t-test comparing equal and selfish allocators | Experiment 1: t(24) = 8.34, p < 0.001, d = 1.67; Experiment 2: t(26) = 8.74, p < 0.001, d = 1.68. |
| **Feelings Function Parameters** (see model comparison results in the table below) | |
| Selfishness aversion parameter | Experiment 1: β = -1.67±0.32 CI = [-2.3, -1.0], t_24_ = -5.1, P < 0.0001.  Experiment 2: β = -0.88±0.33 CI = [-1.53, -0.23], t_27_ = -2.7, P = 0.013. |
| Inequality aversion parameter | Experiment 1: β = -0.71±0.20 CI = [-1.1, -0.31], t_24_ = -3.5, P < 0.01.  Experiment 2: β = -1.1±0.17 CI = [-1.4, -0.75], t_27_ = -6.3, P < 0.0001. |
| Selfishness x Endowment parameter | Experiment 1: β = -0.45±0.10 CI = [-0.65, -0.25], t_24_ = -4.4, P < 0.001.  Experiment 2: β = -0.35±0.11 CI = [-0.56, -0.13], t_27_ = -3.2, P < 0.01. |
| 50% stick parameter | Experiment 1: β = 0.61±0.14 CI = [0.34, 0.88], t_24_ = 4.51, P < 0.001.  Experiment 2: β = 0.67±0.18 CI = [0.32, 1.0], t_27_ = 3.8, P < 0.001. |
| **Observer’s decisions to punish are a function of both selfishness aversion and inequality aversion** | |
| Repeated-measures ANOVA: frequency of punishment | Experiment 1: F(2,48) = 46.12, p < 0.001, η_p_^2^ = 0.66.  Experiment 2: F(2,52) = 177.75, p < 0.001, η_p_^2^ = 0.87. |
| Repeated-measures ANOVA: amount of punishment | Experiment 1: F(2,48) = 107.70, p < 0.001, η_p_^2^ = 0.82.  Experiment 2: F(2,52) = 144.38, p < 0.001, η_p_^2^ = 0.85. |
| Paired-samples t-test comparing selfish and generous allocators: frequency of punishment | Experiment 1: t(24) = 7.01, p < 0.001, d = 1.40.  Experiment 2: t(26) = 13.22, p < 0.001, d = 2.54. |
| Paired-samples t-test comparing selfish and generous allocators: amount of punishment | Experiment 1: t(24) = 11.27, p < 0.001, d = 2.25.  Experiment 2: t(26) = 12.12, p < 0.001, d = 2.33. |
| Paired-samples t-test comparing selfish and equal allocators: frequency of punishment | Experiment 1: t(24) = 7.19, p < 0.001, d = 1.44.  Experiment 2: t(26) = 19.28, p < 0.001, d = 3.71. |
| Paired-samples t-test comparing selfish and equal allocators: amount of punishment | Experiment 1: t(24) = 11.21, p < 0.001, d = 2.24.  Experiment 2: t(26) = 12.41, p < 0.001, d = 2.39. |
| Paired-samples t-test comparing generous and equal allocators: frequency of punishment | Experiment 1: t(24) = 1.06, p = 0.30, d = 0.21. Experiment 2: t(26) = 1.53, p = 0.14, d = 0.29. |
| Paired-samples t-test comparing generous and equal allocators: amount of punishment | Experiment 1: t(24) = 0.41, p = 0.68, d = 0.08.  Experiment 2: t(26) = 0.52, p = 0.61, d = 0.10. |
| **Punishment Function Parameters** (see model comparison results in the table below) | |
| Selfishness aversion parameter | Experiment 1: β = 2.5±0.18 CI = [2.1, 2.8], t_24_ = 14, P < 0.0001.Experiment 2: β = 1.62±0.14 CI = [1.15, 2.10], t_27_ = 6.8, P < 0.0001. |
| Inequality aversion parameter | Experiment 1: β = 0.26±0.13 CI = [0.002 0.52], t_24_ = 2.0, P = 0.06. Experiment 2: β = 1.03±0.11 CI = [0.81, 1.25], t_27_ = 9.2, P < 0.0001. |
| Selfishness x Endowment parameter | Experiment 1: β = 0.32±0.1 CI = [0.14 0.50], t_24_ = 3.5, P < 0.01Experiment 2: β = 0.66±0.15 CI = [0.36, 0.95], t_27_ = 4.4, P < 0.001. |
| 50% stick parameter | Experiment 1: β = -0.6±0.09 CI = [-0.79, -0.44], t_24_ = -6.8, P < 0.0001 ; Experiment 2: β = -0.092±0.076 CI = [-0.24, 0.06], t_27_ = -1.2, P = 0.24. |
| **Observers’ Affective Responses Are Related to Their Punishment** | |
| Mean correlation between feelings and punishment in blocks 2 and 4 of Experiment 1 | r = -0.72, significantly different from zero: t(24) = -17.19, p < 0.001, d = -3.44. |
| Correlation between predicted feelings and observed punishment | Experiment 1🡪 2 : r= -0.75±0.04, CI = [-0.84 -0.66], t(24)= -17.2, p < 0.0001  Experiment 2 🡪 1: r= -0.68±0.05, CI = [-0.79 -0.57], t(27) = -12.7, p < 0.0001 |
| Correlation between predicted punishment and observed feelings | Experiment 1🡪 2: r= -0.68±0.05, CI = [-0.78 -0.57], t(24) = -13.1, p < 0.0001  Experiment 2 🡪 1: r= -0.57±0.08, CI = [-0.74 -0.40], t(24) = -6.8, p < 0.0001 |
| **Out-of-Sample Prediction** |  |
| Relationship between predicted feelings and observed feelings | r^2^ = 0.53, slope vs 1: β = 0.96 ± 0.07, t(52) = -0.62, p = 0.54, constant vs 0: β = -0.02 ± 0.04, t(52) = -0.42, p = 0.68 |
| Relationship between predicted punishment and observed punishment | r^2^ = 0.61, slope vs 1: β = 0.93 ± 0.05, t(52) = -1.4, p = 0.17, constant vs 0: β = 0.03 ± 0.05, t_27_ = 0.64, p = 0.52 |
| **Differences in How Selfishness Aversion and Inequality Aversion Impact Feelings and Actions** | |
| Interaction between response type and social value | F(1,50) = 9.9, p < 0.01, |
| Interaction between response type and experiment | F(1,50) = 3.6, p = 0.06, |
| effect of response | F(1,50) = 12.5, p < 0.0001, |
| effect of social value | F(1,50) = 12.2, p < 0.01, |
| effect of experiment | F(1,50) = 0.045, p = 0.83, |
| interaction between social value and experiment | F(1,50) = 0.52, p = 0.47, |

**Table G**. Results of the statistical tests reported in the main text after exclusion of suspicious participants.

**Observers’ feel better about punishing allocators when those decisions align with their feelings about the allocation.**

The different extents to which inequality aversion and selfishness aversion impact feelings and action will at time result in discrepancies between how people feel about what they observe and whether they attempt to change the status quo. We hypothesized that when such discrepancies occur participants would not feel as good about their decisions to punish.

To test this, we performed linear regressions for each participant that aim to account for participants’ *feelings* *about their* *own punishment decisions* from (i) their *feelings about the allocator’s decision*, (ii) the amount they punished, and (iii) the interaction between the two. This exercise required that for each trial we knew how the participant felt in response to the allocator’s decision, whether and by how much they decided to punish and how they felt about their own decision. In Experiment 1 we indeed had 2 blocks where participants indicated all these variables (blocks 2,4). In experiment 2, however, participants rated how they felt about the allocators’ decisions on two of the blocks (1,3) and made punishment decisions on the other two blocks (2,4). Thus, we used our feelings function which was based on data from Experiment 2 to estimate how participants felt about the allocator’s decision on the punishment trials in Experiment 2 and entered these model-predicted feelings into the regression. We also controlled for the endowment amount which was added as an additional variable.

The results revealed a significant interaction between participants’ feelings about the allocation and the amount they punished (Experiment 1: Mean β = -0.31, SD = 0.21, t(31) = -8.1, p < 0.001, 95% CIs [-0.38, -0.23]; Experiment 2: Mean β = -0.29, SD = 0.15, t(34) = - 2.05, p = 0.048, 95% CIs [-0.58, -0.003]). The interaction was due to participants feeling better about punishing when they felt negatively about the allocation compared to when they felt positively about it.


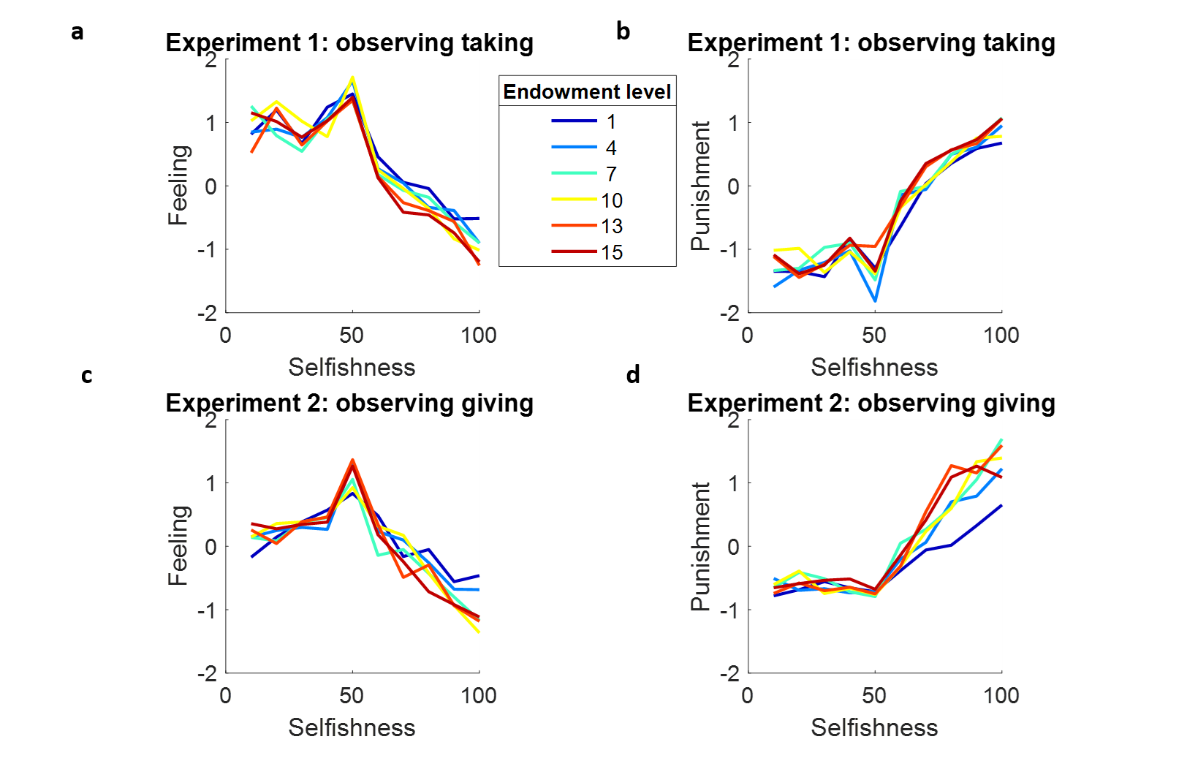


**Fig A. Interaction between endowment and selfishness.** The x-axis represents the observed selfishness and the y-axis feelings (a and b) and punishment decisions (c and d). Each color represents an endowment level, from low (blue, 1) to high (dark red, 15). As can be observed selfishness effected feeling and punishments more for high endowments than low endowments.

******Fig B. Cross-validation of the feeling model.** Each scatter plot corresponds to a participant. The y-axis corresponds to the actual data of one experiment and the x-axis to the predictions of that data from the winning model of the other experiment. The dashed black line corresponds to the y=x line, along which dots would ideally be aligned for perfect validation. The red is the regression line.

******Fig C. Cross-validation of the punishment model.** Each scatter plot corresponds to a participant. The y-axis corresponds to the actual data of one experiment and the x-axis to the predictions of that data from the winning model of the other experiment. The dashed black line corresponds to the y=x line, along which dots would ideally be aligned for perfect validation. The red is the regression line.

**References**

1. Van Lange PAM. The pursuit of joint outcomes and equality in outcomes: An integrative model of social value orientation. J Pers Soc Psychol. 1999;77: 337–349. doi:10.1037/0022-3514.77.2.337

2. Graham J, Nosek BA, Haidt J, Iyer R, Koleva S, Ditto PH. Mapping the Moral Domain What Is the Moral Domain? J Pers Soc Psychol. 2011;101: 366–385.

3. Beck AT, Steer RA, Ball R, Ranieri WF. Comparison of Beck depression inventories -IA and -II in psychiatric outpatients. J Pers Assess. 1996;67: 588–597. doi:10.1207/s15327752jpa6703_13

4. Ashton MC, Lee K. The HEXACO-60: A short measure of the major dimensions of personality. J Pers Assess. 2009;91: 340–345. doi:10.1080/00223890902935878
